# Genetic variation and expression changes associated with molybdate resistance from a glutathione producing wine strain of *Saccharomyces cerevisiae*

**DOI:** 10.1371/journal.pone.0180814

**Published:** 2017-07-06

**Authors:** Francesco Mezzetti, Justin C. Fay, Paolo Giudici, Luciana De Vero

**Affiliations:** 1 Department of Life Sciences, University of Modena and Reggio Emilia, Reggio Emilia, Italy; 2 Department of Genetics and Center for Genome Sciences and Systems Biology, Washington University, St. Louis, Missouri, United States of America; Tulane University Health Sciences Center, UNITED STATES

## Abstract

Glutathione (GSH) production during wine fermentation is a desirable trait as it can limit must and wine oxidation and protect various aromatic compounds. UMCC 2581 is a *Saccharomyces cerevisiae* wine strain with enhanced GSH content at the end of wine fermentation. This strain was previously derived by selection for molybdate resistance following a sexual cycle of UMCC 855 using an evolution-based strategy. In this study, we examined genetic and gene expression changes associated with the derivation of UMCC 2581. For genetic analysis we sporulated the diploid UMCC 855 parental strain and found four phenotype classes of segregants related to molybdate resistance, demonstrating the presence of segregating variation from the parental strain. Using bulk segregant analysis we mapped molybdate traits to two loci. By sequencing both the parental and evolved strain genomes we identified candidate mutations within the two regions as well as an extra copy of chromosome 1 in UMCC 2581. Combining the mapped loci with gene expression profiles of the evolved and parental strains we identified a number of candidate genes with genetic and/or gene expression changes that could underlie molybdate resistance and increased GSH levels. Our results provide insight into the genetic basis of GSH production relevant to winemaking and highlight the value of enhancing wine strains using existing variation present in wine strains.

## Introduction

Oxidation during wine production is an important source of off-flavors [1,2]. Glutathione (GSH) is a strong antioxidant and may improve wine quality through preservation of aromatic compounds produced during fermentation and prevention of off-flavors generated by oxidation [3]. Moreover, GSH could contribute to the reduction of sulfur dioxide, which is used to prevent wine spoilage but can have a negative impact on consumer preference and flavor [4]. Accordingly, there is strong interest in developing novel wine yeast strains with enhanced GSH production [5].

Evolutionary engineering is a powerful approach to strain development when genetically modified yeast cannot be used in wine production, as is the case for many wine markets [6,7]. Evolutionary engineering combines both traditional breeding with selection for desired characteristics. Selection can act on either *de novo* mutations or naturally occurring variation. While selection assists in obtaining desired strains the process can be lengthy if taken through multiple rounds of screening/selection and many traits are not directly selectable at the population level. Nevertheless, this approach has been successfully used to generate wine yeast strains with improved properties, such as enhanced substrate utilization, tolerance to fermentation conditions and resistance to toxic compounds [8–10].

An important factor in evolutionary engineering is the ability to select for a desirable trait such as GSH levels. In previous work [11], a high GSH producing strain was derived by selection for molybdate resistance. Because molybdate is a toxic sulfate analogue, it enters yeast cells through high-affinity sulfate permeases [12–14]. Under normal conditions sulfate enters a cell and is processed through the sulfur assimilation pathway to produce homocysteine, a precursor to cysteine and GSH production. Thus, selection for molybdate resistance may alter the sulfur assimilation and GSH production pathway to either inhibit its entry into the cell or increase resistance through the formation of GSH-metal complexes that are sequestered or removed from the cell [15–18].

Starting with a wine strain 21T2 (equivalent to UMCC 855), Mezzetti et al. [11] derived multiple molybdate resistant strains with elevated GSH levels using an evolutionary engineering approach. Although the mechanism of molybdate resistance and GSH production are not known, the most likely mechanism is through activating of the cells' metal response including sulfur assimilation and GSH biosynthesis [11].

In the present work, we characterized genetic and gene expression changes associated with molybdate resistance phenotypes present in the GSH producing strain UMCC 2581. Because the evolved GSH-producing strain UMCC 2581 was derived from UMCC 855 through sporulation and selection under a specific selective pressure, its high level of molybdate resistance could be due to selection on *de novo* mutations that occurred during the process or on segregation of variants present in the heterozygous state in UMCC 855. To examine these possibilities we first measured molybdate resistance among segregants of the parental UMCC 855 strain and found four different phenotypic clusters. Using these strains, we mapped molybdate resistance using bulk segregant analysis to two loci. To examine any additional genetic changes that might have occurred during selection we sequenced the genomes of the parental (UMCC 855) and evolved (UMCC 2581) strains and measured gene expression differences between them in the presence of molybdate. Our results point to a number of potential candidate genes and expression changes implicated in molybdate resistance and GSH production.

## Materials and methods

### Yeast strains and phenotyping

The *Saccharomyces cerevisiae* strains used in this study (Table 1) include a diploid wine strain (UMCC 855) along with a derivative (UMCC 2581) selected for high GSH levels by Mezzetti et al. [11]. These strains are available from the Unimore Microbial Culture Collection (University of Modena and Reggio Emilia- Reggio Emilia- Italy). For genetic mapping, we generated monosporic clones of UMCC 855. An overnight culture of UMCC 855 was grown at 28°C on YPDA (1% yeast extract, 2% peptone, 2% dextrose, 2% agar), resuspended in 3 mL of 1% potassium acetate and incubated at 28°C overnight with shaking at 300 rpm to induce sporulation. The sporulated cells were diluted 2-fold with a solution of 10 mg/mL Zymolyase (20T, Fisher Scientific) and incubated for 1 h at 28°C and then spotted onto YPDA plates. Tetrads were dissected using a micromanipulator (Singer Instruments MSM System 200) and a total of 69 monosporic clones (MCs) were recovered.

**Table 1:** Parental and evolved *Saccharomyces cerevisiae* strains.

UMCC^a^ code	Name	Genotype^b^	Description	References
**UMCC 855**	21T2	*MAT a/MAT α*; *ho-*; diploid	Laboratory yeast strain selected for its oenological aptitude.	[11,19,20]
**UMCC 2581**	Mo21T2-5	*MAT a/MAT α*; *ho-*; diploid	Evolved yeast strain from UMCC 855, high GSH producer.	[11]

^a^Unimore Microbial Culture Collection (UMCC), University of Modena and Reggio Emilia- Reggio Emilia (Italy).

^b^Naturally occurring *ho-* was inferred based on the production of *MATa* and *MATα* progeny following tetrad dissection.

Molybdate resistance was measured by growth over a range of concentrations. Strains were plated on YNB (0.17% yeast nitrogen base without amino acids and ammonium sulfate, DIFCO, Detroit, MI, 2% agar, 2% glucose, 100 μM ammonium sulfate, Sigma-Aldrich, St. Louis, MO, 1% yeast extract) supplemented with molybdate Mo(VI) at concentrations of 0 (control), 1.0, 2.5, and 5.0 mM to evaluate molybdate resistance. Colony growth and color after 4 days of growth at 28°C was used to group the MCs into four phenotypic clusters: two resistant clusters (9 MCs for each clusters), a sensitive cluster (37 MCs) and a cluster with intermediate phenotypes (14 MCs). The two resistant clusters were named “Resistant-Parental” and “Resistant-Evolved” based on their phenotypic similarity to UMCC 855 and UMCC 2581, respectively (S1 Fig). The Resistant-Parental group was able to grow at 2.5 mM Mo(VI) but not at 5.0 mM Mo(VI) and showed white or light blue colonies, the Resistant-Evolved group was able to grow at 2.5 mM Mo(VI) and often at 5.0 mM Mo(VI) and showed dark blue colonies (S1 Fig). The sensitive group was unable to grow on molybdate at any concentration. The intermediate group included the MCs which showed a different phenotype compared to the other two resistant groups and were able to grow at 1.0 mM Mo(VI) or higher.

### Bulk segregant analysis

**Fig 1:**
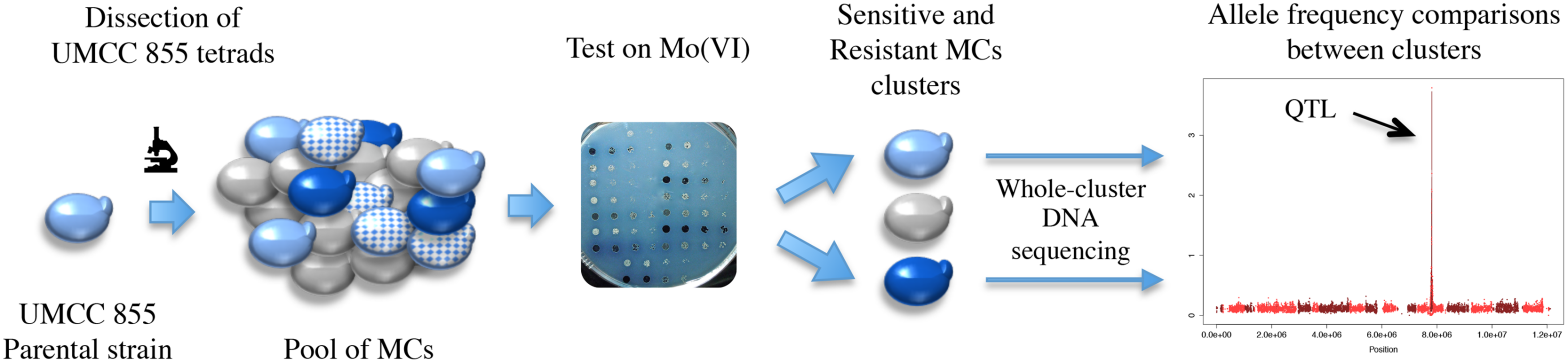
Bulk segregant mapping strategy. The parental strain UMCC 855 was sporulated and 69 monosporic clones (MCs) were grouped into phenotypic clusters based on their growth on molybdate-containing plates. DNA was extracted from a pool of strains from each cluster and sequenced to identify variants associated with molybdate resistance.

Molybdate resistance was mapped using bulk segregant analysis [21] (Fig 1). However, rather than only comparing genotype frequencies of resistant and sensitive segregants we compared genotype frequencies of the two resistant and one sensitive phenotypic cluster. Genomic DNA was extracted from each pool of segregants (ZR fungal/bacterial DNA miniprep, Zymo Research) and genome sequencing was performed using the Ion Torrent ProtonTM platform (Life Technologies) by the Genomics Core Facility at Saint Louis University School of Medicine. Genomic DNA was also extracted and sequenced for the parental and evolved strain. Sequence reads were mapped to the *S*. *cerevisiae* reference genome, S288c [22], using BWA (Burrows-Wheeler Aligner) [23]. Duplicate sequences were removed using PicardTools 1.114 (http://broadinstitute.github.io/picard) and single nucleotide polymorphisms (SNPs) and insertion/deletions (InDels) were called using the Genome Analysis Toolkit (GATK v3.4–46) [24]. We obtained an average of 26,863 SNPs and 955 InDels from and average of 7,031,003 reads across each of the three pools (S1 Table).

To map molybdate resistance we first identified heterozygous sites in the parental strain and then tested whether these SNPs differed in allele frequency between phenotypic clusters. Heterozygous sites identified in the parental strain were filtered for allele frequency ranging from 0.25–0.75 and coverage of at least 20 reads using a perl script (S1 File). After filtering, a total of 18,047 heterozygous sites were examined for allele frequency differences using the log10 of the odds ratio (LOD) of the two frequencies in each cluster. A LOD score greater than 3 was chosen as a cutoff to call QTL peaks and the width of each peak was determined by the LOD score decreasing by 1 unit.

Candidate genes were identified by annotating SNPs and InDels within QTL. Heterozygous SNPs and InDels within the parental strain UMCC 855 were annotated using snpEff software [25] (http://snpeff.sourceforge.net). Variants within protein-coding regions were annotated by whether they altered the protein sequence or not. Candidate variants were further examined to ensure high quality variants by examining the read alignments, alignment quality, coverage and variant quality.

To identify any *de novo* mutations in UMCC 2581 we compared its genome to the parental strain UMCC 855. To identify any large regions with copy-number variations (CNVs), the average sequencing coverage across 1000 bp windows were calculated using IGVtools [26]. To identify *de novo* SNPs and InDels we filtered out all sites called heterozygous in UMCC 855 or carrying the same variant in both UMCC 855 and UMCC 2581.

### RNA-sequencing and differential gene expression analysis

Gene expression differences between the parental and evolved strain were measured by RNA-sequencing. Three replicate cultures were used for the parental strain and a single replicate was used for the evolved strain. The strains were grown in 100 mL of synthetic must (SM, S2 Table) in Erlenmeyer flask prepared according to Giudici and Kunkee [27]. Cells were harvested at three quarters of their exponential growth period, measured by optical density at 600 nm at hourly intervals. Cells were centrifuged and the pellet immediately frozen in liquid nitrogen and stored at -80°C. RNA was extracted with hot acidic phenol [28], mRNA was purified using Ambion Dynabeads mRNA Direct Micro Kit (Life Technologies), and RNA-Seq libraries were prepared by the Genomics Core Facility at Saint Louis University School of Medicine using the Ion Total RNA-seq kit v2 (Life Technologies) and sequenced using the Ion Torrent ProtonTM platform (Life Technologies). Bowtie 2 [29] was used to align reads to the reference genome S288c, Picard tools (1.114) was used to eliminate duplicate reads and DESeq [30] was used to identify differentially expressed genes. The DESeq analyses used the variance among the parental replicates to calculate p-values by assuming the variance among replicates is the same for the two strains. Genes with a false discovery rate (FDR) lower than 0.05 were considered differentially expressed. The gene ontology analysis was performed by using the on-line tool GO Term Finder (http://www.yeastgenome.org/cgi-bin/GO/goTermFinder.pl) from the SGD database. The statistical analysis of the GO term “molecular function” was performed using the BiNGO plug-in [31] in Cytoscape (http://www.cytoscape.org).

### Accession number

All DNA sequencing and RNA-seq data are available from the NCBI Sequence Read Archive (SRA, https://www.ncbi.nlm.nih.gov/sra/) with accession number SRP094104.

## Results

### Mapping molybdate resistance through bulk segregant analysis

The evolved molybdate resistant strain UMCC 2581 grows well at 2.5 mM Mo(VI) and has dark colonies, whereas the parental strain UMCC 855 does not grow well and has light colored colonies (Table 2). Because UMCC 2581 was derived by sporulation of the parental strain as well as selection for molybdate resistance, the higher resistance of the evolved strain could be due to *de novo* mutations that arose during the selection process or segregation of heterozygosity present in the parental strain. To determine whether heterozygosity in the parental strain influences molybdate resistance we sporulated the parental strain and examined molybdate resistance of 69 monosporic clones (MCs). We found that 37 (54%) of the MCs were not able to grow on 1.0 mM Mo(VI). Of the remaining MCs, 9 showed molybdate resistance similar to the parental strain (Resistant-Parental), another 9 showed molybdate resistance similar to the evolved strain (Resistant-Evolved) and the remaining 14 MCs showed intermediate levels of molybdate resistance. Thus, the parental strain carries genetic variation affecting molybdate resistance.

**Table 2:** Clustering of the segregants obtained from the strain UMCC 855.

Strains and clusters	Description	Growth on YNB with Mo(VI) 2.5 mM	
		Colony color	Resistance
UMCC 855	Parental strain	White/Light blue	Low/Intermediate
UMCC 2581	Evolved strain	Dark blue	High
Cluster Resistant-Parental	Pool of 9 segregants	White/Light blue	Low/Intermediate
Cluster Resistant-Evolved	Pool of 9 segregants	Dark blue	High
Cluster Sensitive	Pool of 37 segregants	No growth	Sensitive

To map the genetic basis of molybdate resistance we used bulk segregant analysis [21]. Genomic DNA libraries were constructed from three pools of MCs based on molybdate resistance: sensitive, Resistant-Parental and Resistant-Evolved. To identify heterozygous sites within the parental strain we also generated a genomic DNA library of UMCC 855. Sequencing of these libraries yielded genome coverage of 133-fold for the parental strain and 66-, 78-, and 104-fold for the sensitive, Resistant-Parental and Resistant-Evolved pools, respectively. For the parental strain we identified 13,687 heterozygous single nucleotide polymorphisms (SNPs) and 4,360 heterozygous insertion/deletions (InDels) polymorphisms. Using these polymorphisms we calculated the frequency of these variants in each of the three MCs pools and identified variants with different frequencies using the log of the odds ratio (LOD) scores.

**Fig 2:**
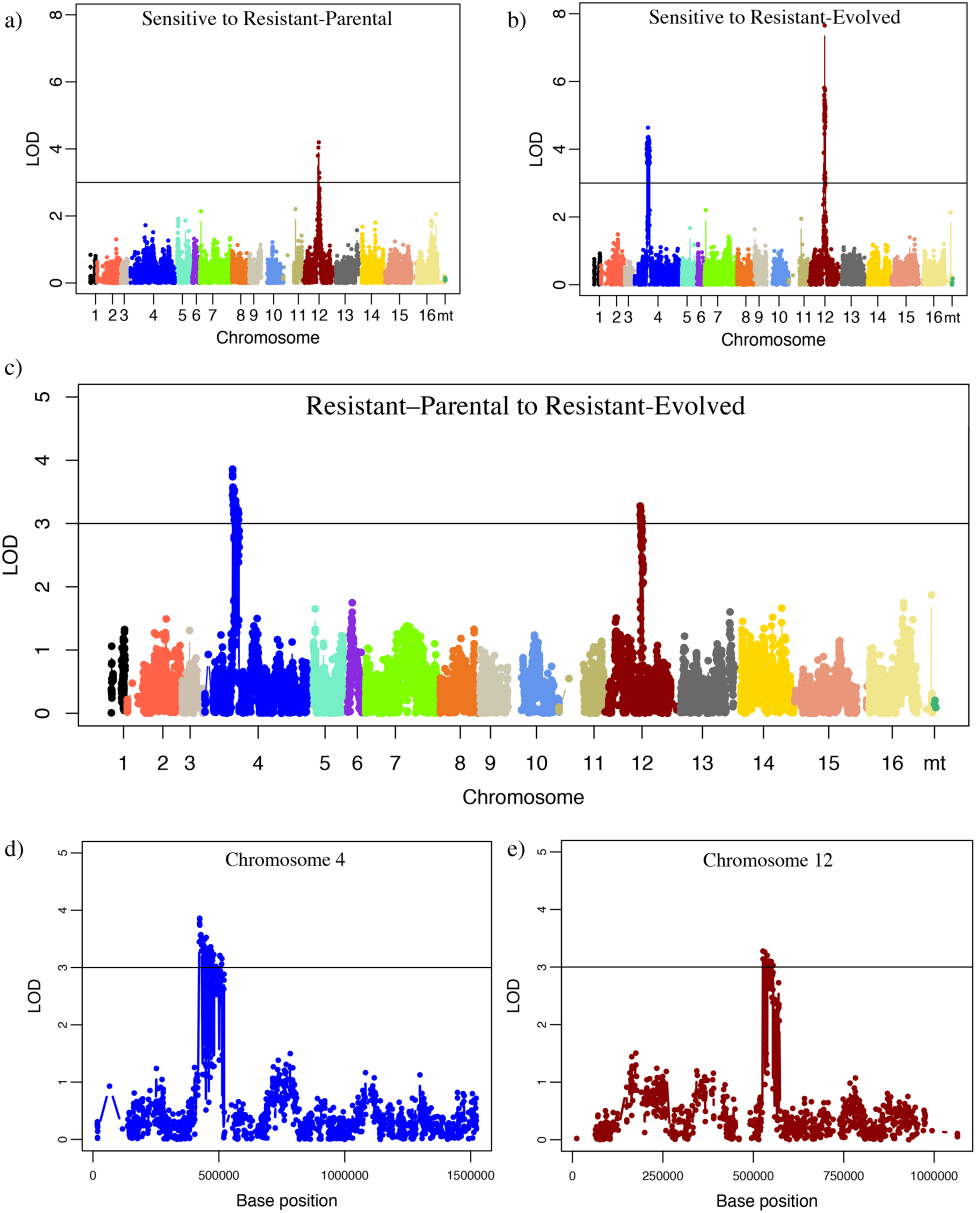
QTL mapping of molybdate resistance. Molybdate resistance associations are shown by the log-odds ratio (LOD) of allele frequency differences between phenotypic clusters: Sensitive to Resistant-Parental (a), Sensitive to Resistant-Evolved (b), and Resistant-Parental to Resistant-Evolved (c). Chromosomes are displayed on the x-axis and LOD score for each variant on the y-axis. A LOD score of 3 was chosen as a QTL threshold (black line). d) LOD plot of the chromosome 4 QTL for Resistant-Parental to Resistant-Evolved. e) LOD plot of the chromosome 12 QTL for Resistant-Parental to Resistant-Evolved.

Comparing each of the two resistant groups to the sensitive group revealed a large peak in the LOD score on chromosome 12 (Fig 2a and 2b) and a second peak on chromosome 4 for the sensitive to Resistant-Evolved comparison. Comparison of the two resistant groups to each other revealed two peaks: one peak on chromosome 4 and one on chromosome 12 (Fig 2c, 2d and 2e). These results suggest a major factor contributing to molybdate resistance occurs on chromosome 12 and a smaller effect, which distinguishes the two resistant groups, on chromosome 4.

### Identification of candidate genes

To identify candidate genes within the mapped regions, we examined gene function and genes with heterozygous sites within the parental strain. For each peak, we defined the region of interest by the flanking locations where the LOD score decreased 1 unit lower than the peak. This definition yielded two largely overlapping regions on chromosome 12 when comparing the sensitive group to the Resistant-Parental group (512,000 to 560,000 bp) and the Resistant-Evolved group (527,000 to 572,000 bp) giving a common region from 527,000 to 560,000 bp. A second peak on chromosome 4 (422,000 to 521,000 bp) was found in the sensitive to Resistant-Evolved comparison. Finally, a linkage region on chromosome 4 (422,000 to 521,000 bp) and chromosome 12 (527,000 to 571,000 bp) was found for the Resistant-Parental to Resistant-Evolved mapping. For each region, we identified genes with annotated functions potentially relevant to oxidative stress, metal resistance or GSH. Only the common region was used for candidate gene analysis on chromosome 12. Across the two regions there are 85 annotated genes. Of these genes, nine have functions with plausible roles in molybdate resistance and 57 have heterozygous altering variants in the parental strain but homozygous variants in the evolved strain (Table 3).

**Table 3:** Numbers of relevant genes and variants in the QTL regions.

Region	ORF	Genes potentially relevant to the resistant phenotype	Genes with variants heterozygous in UMCC 855 and homozygous in UMCC 2581
Chromosome 4	56	6	36
Chromosome 12*	29	3	21
Total	85	9	57

* The union of the regions identified in the different clusters comparison are considered.

### Identification of new mutations

**Fig 3:**
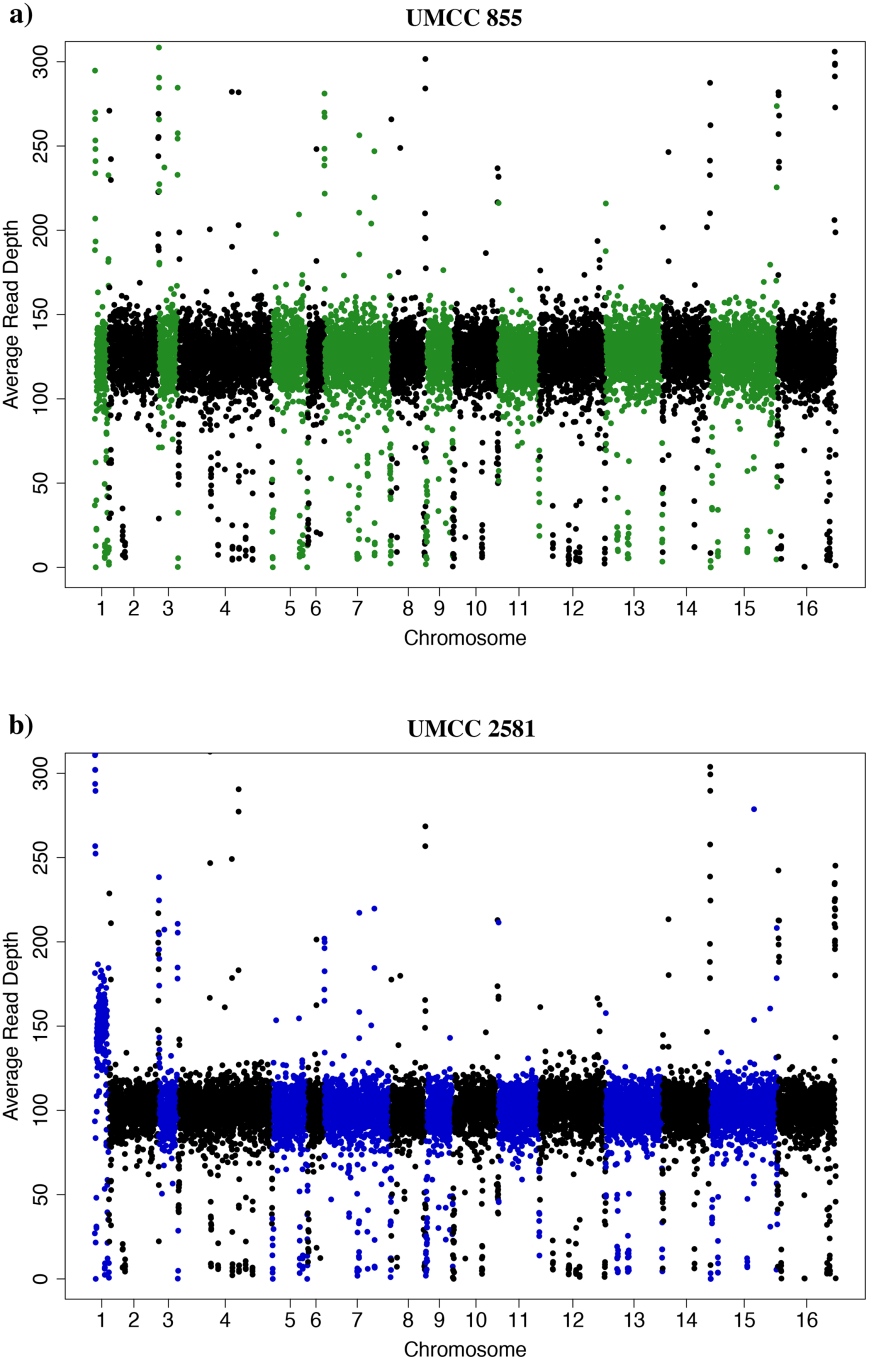
Chromosomal aneuploidy determined by whole-genome sequencing coverage. The average sequencing coverage across the genome is shown using a sliding window of 1000 bp. Each window is shown by a dot and colors (green or blue and black) alternate between chromosomes. Coverage across the UMCC 855 parental strain genome (a) and the UMCC 2581 evolved strain genome (b).

The evolved strain may not only exhibit high molybdate resistance due to re-assortment of heterozygosity present in the parental strain but may also carry *de novo* mutations that occurred during the selection process. To identify new mutations in the evolved strain we sequenced its genome and compared it to the parental strain genome. We found 61 SNP and 847 InDels present in the evolved strain but absent from the parental strain. To catalogue copy-number variation (CNV) we used the depth of sequencing coverage across the genome (Fig 3). While the parental strain exhibited a normal chromosomal set (Fig 3a), the evolved strain had higher coverage of chromosome 1 indicative of aneuploidy (Fig 3b). The read depth of chromosome 1 was 1.5-fold greater than the median of the strain, suggesting an extra-copy of this chromosome in UMCC 2581.

### Gene expression differences between the parental and evolved strains

To examine potential mechanisms of higher GSH production and resistance to Mo(VI), we assessed gene expression levels of the evolved and parental strains. Gene expression was measured at three quarters of the way through the exponential phase of growth in synthetic must. This point was chosen as it occurs before the major transcriptional reprogramming that occurs upon entry into stationary phase [32,33], and gene expression levels are expected to be relatively stable and indicative of the capacity to produce GSH during fermentation. At this time-point we extracted RNA from three replicate cultures of the parental strain and a single culture of the evolved strain and measured gene expression levels by RNA-sequencing.

**Fig 4:**
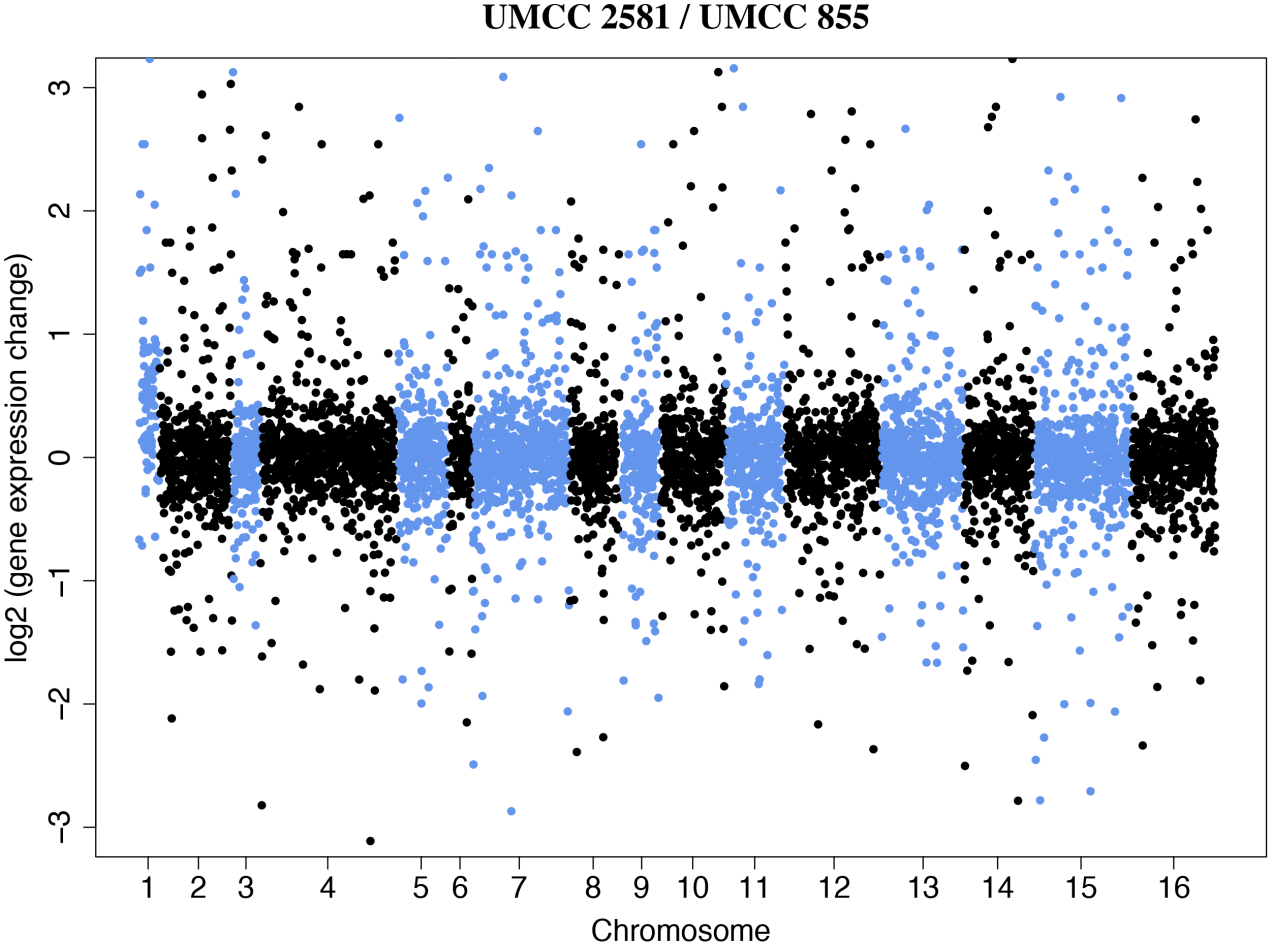
Expression profiles of UMCC 2581 compared to UMCC 855. Each dot represents the fold-change (log2) in gene expression between the two strains (UMCC 2581/UMCC 855). The dots colors (light blue and black) alternate between chromosomes.

**Fig 5:**
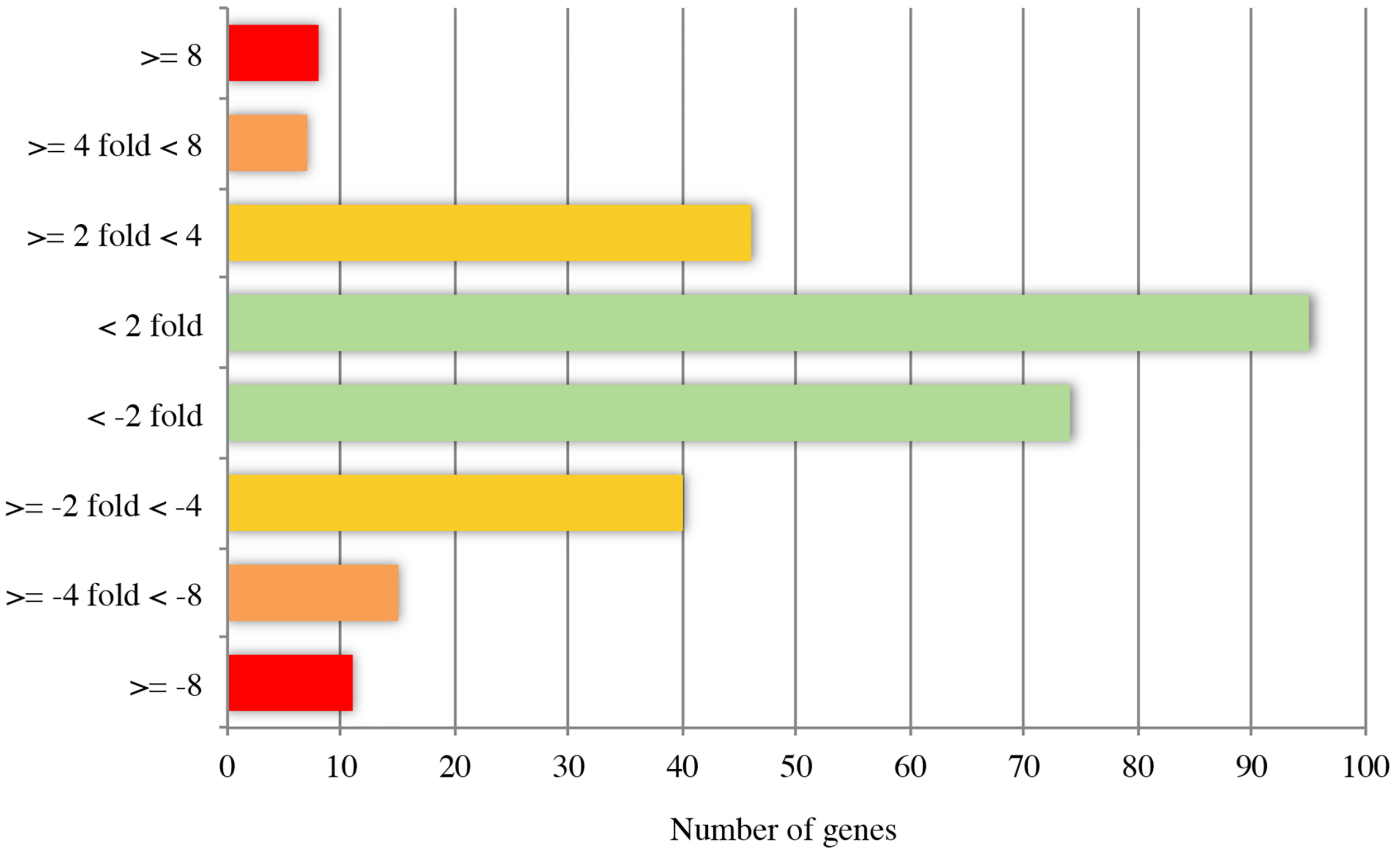
Distribution of genes differentially expressed between UMCC 855 and UMCC 2581. The values of the x-axis represent the number of genes significantly (FDR < 0.05) up- or down-regulated in UMCC 2581 compared to UMCC 855. The number of genes are binned into four color-coded groups by their fold-change (y-axis).

The transcriptomes of the UMCC 2581 evolved strain and the UMCC 855 parental strain were first compared in relation to their genomic position (Fig 4). Consistent with chromosome 1 aneuploidy we found higher average expression of the genes on chromosome 1 of the evolved strain (0.57) compared to the average expression of all other chromosomes taken together (0.04). For all subsequent analyses we removed the 47 differentially expressed genes present on chromosome 1. After removing these genes on chromosome 1 we found 296 genes differentially expressed between the two strains (FDR < 0.05, Fig 5 and S3 Table). Of the 161 genes with higher expression in evolved strain, 66 genes had a two-fold or greater difference and out of 135 genes with lower expression, 61 genes had a two-fold or greater difference. Our results indicate that a small fraction of the genes in the genome show large differences in gene expression.

Among the top 10% of genes that were strongly up-regulated in UMCC 2581 (16 genes, S3 Table), we found two permease genes (*DIP5* and *GNP1*) involved in transport of GSH precursor amino acids (cysteine, methionine, glutamate and glycine) and *SUL1* involved in sulfate assimilation. On the contrary, among the top 10% of genes strongly down-regulated in UMCC 2581 (14 genes), we did not find any genes potentially involved in the resistant phenotype.

**Fig 6:**
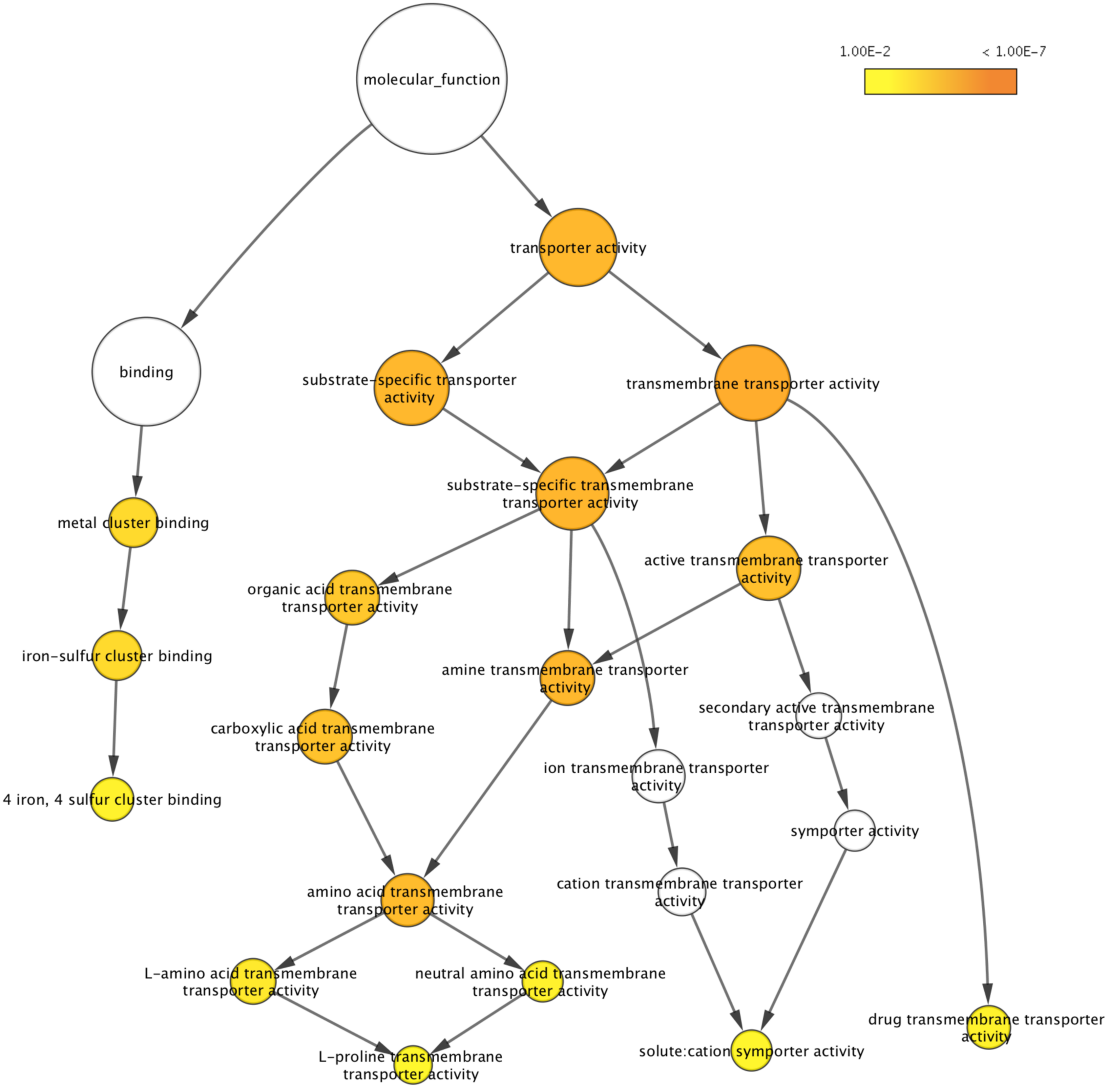
Gene ontology (GO) terms associated with genes up-regulated in UMCC 2581 compared to UMCC 855. Associated GO terms for molecular function are shown by nodes (circles) and related to one another using BiNGO. The node size corresponds to the number of proteins that are assigned to individual terms. Terms with a p-value below 0.01 are shown in yellow and a darker color represents a lower p-value (orange < 1.00E-7). White nodes are not significantly over-represented; they are included to show the colored nodes in the context of the GO hierarchy.

Using the differentially expressed genes we search for enriched gene ontology (GO) terms. We identified significant GO terms (FDR < 0.05) for biology processes (S4 Table), molecular function (Table 4 and S5 Table) and cellular component (S6 Table). For genes up-regulated in the evolved strain we found enrichment of genes in anion and amino acid transport processes and a corresponding enrichment of genes with anion and amino acid transport functions and localization to the cell periphery and plasma membrane. The graphical representation of the molecular function GO terms (Fig 6) showed that within genes with transporter activity, amino acid transport functions were particularly enriched. As shown in Table 4, many of the genes present in all the enriched classes are involved in amino acid transport. Noteworthy, seven out of nine genes with amino acid transmembrane transporter activity are genes related to GSH precursor amino acids. In particular, Dip5p mediates high-affinity transport of L-glutamate but it is also a transporter for glycine; *YCT1* and *MUP3* encode, respectively, a high-affinity cysteine transporter and a low affinity methionine permease; Gnp1p transports both the amino acids, cysteine and methionine; Agp1p is a low affinity amino acid permease with broad substrate range; and Gap1p is a general amino acid permease. Finally, even though not a transporter for amino acids, the high affinity sulfate permease, encoded by *SUL1*, is involved in methionine and cysteine biosynthetic process by the sulfate assimilation pathway.

**Table 4:** Selected Gene Ontology—Function.

Gene Ontology term	Number of genes	p-value	Genes annotated to the term^a^
transmembrane transporter activity	28	2.81E-06	*FUR4*, *CTP1*, *VBA2*, ***SUL1***, *PHO89*, ***AGP1***, *SNQ2*, *YCF1*, *HXT7*, ***GNP1***, *STL1*, *FCY2*, *HNM1*, *MEP1*, *MPC3*, *DUR3*, ***MUP3***, *HXT5*, *QDR2*, ***GAP1***, ***YCT1***, ***MMP1***, *ATR1*, *HXT2*, *FET3*, *TAT2*, *ENB1*, ***DIP5***
substrate-specific transmembrane transporter activity	27	1.72E-06	*FUR4*, *CTP1*, *VBA2*, ***SUL1***, *PHO89*, ***AGP1***, *YCF1*, *HXT7*, ***GNP1***, *STL1*, *FCY2*, *HNM1*, *MEP1*, *MPC3*, *DUR3*, ***MUP3***, *HXT5*, *QDR2*, ***GAP1***, ***YCT1***, ***MMP1***, *ATR1*, *HXT2*, *FET3*, *TAT2*, *ENB1*, ***DIP5***
anion transmembrane transporter activity	16	3.68E-08	*CTP1*, *VBA2*, ***SUL1***, *PHO89*, ***AGP1***, *YCF1*, ***GNP1***, *HNM1*, *MPC3*, ***MUP3***, ***GAP1***, ***YCT1***, ***MMP1***, *ATR1*, *TAT2*, ***DIP5***
cation transmembrane transporter activity	15	0.00255	*FUR4*, *PHO89*, ***AGP1***, ***GNP1***, *STL1*, *HNM1*, *MEP1*, *DUR3*, ***MUP3***, *QDR2*, ***GAP1***, ***YCT1***, *FET3*, *TAT2*, *ENB1*
carboxylic acid transmembrane transporter activity	13	1.38E-07	*CTP1*, *VBA2*, ***AGP1***, *YCF1*, ***GNP1***, *HNM1*, *MPC3*, ***MUP3***, ***GAP1***, ***YCT1***, ***MMP1***, *TAT2*, ***DIP5***
amino acid transmembrane transporter activity	9	2.78E-05	*VBA2*, ***AGP1***, ***GNP1***, ***MUP3***, ***GAP1***, ***YCT1***, ***MMP1***, *TAT2*, ***DIP5***

Comparison between gene expression levels of the UMCC 2581 and UMCC 855 strains. Selected Gene Ontology (GO) terms for molecular function enriched for up-regulated genes in UMCC 2581 are reported.

^a^In bold are permease genes related to GSH precursor amino acids.

Another set of genes not identified as enriched by GO terms are related to GSH production. *MET5*, *GLT1* and *SER3* were up-regulated in UMCC 2581 and are involved in biosynthesis of methionine and cysteine (*MET5*, sulfite reductase beta subunit), glutamate from glutamine and alpha-ketoglutarate (*GLT1*, NAD(+)-dependent glutamate synthase) and glycine (*SER3*, 3-phosphoglycerate dehydrogenase).

GO term analysis of down-regulated genes showed few associated terms. Only four genes (*THI2*, *THI4*, *THI20*, *THI21*) related to the biological process of metabolism of thiamine were identified as an enriched biological process.

## Discussion

GSH-producing wine yeasts are of great interest due to the important role of GSH in limiting must and wine oxidation and in protecting various aromatic compounds. Evolutionary engineering, involving selection for desirable phenotypes over multiple generations and/or sexual cycles, has been successfully used to improve yeast strains used for wine fermentation without creating genetically modified organisms. In a previous study [11], evolutionary engineering was used to derive an evolved strain (UMCC 2581) with enhanced GSH production by selection for molybdate resistance. In this study, we used bulk segregant analysis, genome sequencing of the parental (UMCC 855) and evolved strain, and transcriptome profiling to identify the genetic and molecular basis of the phenotypic differences between these strains. We find a locus on chromosome 12 has a large effect on molybdate resistance among progeny of the parental strain. However, we also find another locus on chromosome 4 that is associated with the enhanced phenotype (colony color and molybdate resistance) in the evolved strain. Combining the QTL, genomic analysis and transcriptome analysis we identify and discuss below a number of plausible genes and mechanisms of molybdate resistance and GSH levels of the evolved strain.

### Known and potential mechanisms of molybdate resistance and GSH production

There are a number of known pathways that could mediate both molybdate resistance and GSH levels. The relationship between metal uptake and toxicity has been documented in many instances since metal resistant microbial strains often exhibit an ability to prevent or reduce entry of toxic metal species into the cell [34,35]. Among the events conferring resistance to sulfate toxic analogues, a mutation in the high-affinity permeases encoded by the genes *SUL1* and *SUL2* is one of the most probable [12–14]. In this case, the subsequent impaired assimilation of sulfate [36] can lead to altered GSH production. GSH biosynthesis in *S*. *cerevisiae* takes place in two ATP-dependent steps. In the first, cysteine is linked with glutamate by γ-glutamylcysteine synthetase (encoded by *GSH1*) to form γ-glutamylcysteine. In the second step, glycine is added to this intermediate product by glutathione synthetase (encoded by *GSH2*) to form the final product [37,38]. Because the end product of sulfur assimilation is homocysteine, used to generate cysteine, disruption of sulfur assimilation would require import of cysteine or synthesis of cysteine via the methionine salvage pathway. Thus, disruption of sulfur assimilation could lead to altered GSH production, either higher or lower depending on how a cell compensates for disrupted sulfur assimilation.

Another resistance mechanism of the cells could be related to the production of GSH, which is known to have an essential role in the defense against oxidative stress and metal toxicity [14,39]. Several authors have reported that GSH is able to chelate heavy metals by forming complexes (metal-GSH complex) that are actively transported into the vacuole or removed from the cell by specific transporters such as Ycf1p and Gex1p [11,15–18]. Finally, novel or unknown mechanisms could lead to molybdate resistance and enhanced GSH production.

### Candidate genes for molybdate resistance

Through the analysis of the sensitive and two resistant clusters we identified two QTL regions of interest. A single QTL on chromosome 12 was found to be responsible for the difference between the sensitive segregants and the two resistant groups. Within the chromosome 12 QTL we identified 2 genes of interest based on their function and 12 genes of interest based on heterozygous amino acid polymorphisms in the parental strain. The two annotated genes with function potentially related to molybdate resistance were *MDL1* and *HMX1*. *MDL1* encodes a mitochondrial inner membrane ATP-binding Cassette (ABC) transporter and it is required for mitochondrial export of peptides [40]. Experiments carried out by Chloupková and co-workers [41], demonstrated that Mdl1p has a role in resistance to oxidative stress and in particular that null mutant cells were resistant to multiple oxidants. The UMCC 855 sequence of *MDL1* gene did not show any heterozygous amino acid polymorphisms, however, we found a heterozygous frameshift mutation (GT > G) in gene position 1,221 of UMCC 2581 sequence. We hypothesize that this mutation led to an increased resistance to molybdate in UMCC 2581 since it occurred just before the ABC transporter-type domain of the protein presumably resulting in the lost of function of Mdl1p that carried this frameshift.

The second annotated gene within the chromosome 12 QTL, *HMX1*, encodes a heme oxygenase of the endoplasmic reticulum [42]. Besides its function in catalyze the oxidation of heme, it has been recently demonstrated an additional role of Hmx1p in cellular antioxidant protection via transcriptional regulation of antioxidant genes [43]. Collinson and colleagues showed that Hmx1p was induced when yeast cells were exposed to different stresses producing changes in cellular glutathione content and GSH-related antioxidant activities. Two heterozygous polymorphisms were present in the *HMX1* sequence of parental strain but both reference alleles were fixed in evolved strain. The first mutation was a frameshift (A>AAG, S7 Table) that arose almost at the end of the coding sequence in amino acid position 313. The second polymorphism resulted in a missense variation in the amino acid position 110 (Tyr110His), in the heme oxygenase domain of the protein. Thus, we speculate that a fully functional Hmx1p could provide an important contribution in the definition of UMCC 2581 phenotype, inducing GSH-related antioxidant activities.

From the sensitive relative to Resistant-Evolved mapping we identified an additional QTL on chromosome 4 in addition to the chromosome 12 QTL. Among the 85 annotated genes within the two QTL, nine were functionally related to GSH production (*GCV1*), resistance to metals (*KCS1* and *FRE1*) or to oxidative stress (*GRX6*, *MED2*, *NTH1*, *PST2*, *MDL1*, *HMX1*), but only seven of them had amino acid altering variants that differed between the evolved and parental strains (S7 and S8 Tables). The only gene within the two QTL regions with a function related to GSH production was *GCV1* on chromosome 4. *GCV1* encodes for the glycine cleavage T-protein subunit involved in the catabolism of glycine, which is a precursor of GSH [44]. However, no differences between the parental and evolved strain were present in the sequences. Although the vacuole emerges as a major hot-spot for metal detoxification, a number of pathways play a more general role in promoting cell survival under stressful conditions. In this context, the products of *KCS1* on chromosome 4 and *FRE1* on chromosome 12 could be involved in the metal detoxification. The protein encoded by *KCS1* is an inositol hexakisphosphate kinase. The inositol pyrophosphates synthesized by this enzyme is related to a wide range of cellular functions as vacuolar biogenesis, stress response, vesicular trafficking, apoptosis, telomere maintenance and protein phosphorylation [45]. In a recent study, Du and co-workers [46], linked the deletion of *KCS1* gene to the sensitivity of yeast cells to metals, indicating that Kcs1p could be related to metal resistance. In UMCC 855 we observed three heterozygous sites in *KCS1* that cause missense changes. In amino acid position 320 and 560 (Arg320Ser, Phe560Leu) the alternative allele was fixed in the evolved strain, whilst in position 463 (His463Tyr) the reference allele was restored (S8 Table). These missense mutations arose inside and between the two inositol polyphosphate kinase domains and could affect the functionality of the protein. Nevertheless, further analyses are necessary to better understand the putatively effect of these mutations on the studied phenotype. *FRE1* encodes a cell-surface iron and cupric reductase and it is mainly required for iron uptake in yeast strains [47]. Large-scale studies with fre1 null mutant cells displayed a different phenotype with respect to different tested metals: *FRE1* deletion protects against copper by lowering copper import [48], yet it could increase the expression of low-affinity transporters and the accumulation of other metals such as Mn and Co that overwhelm the protective systems in the cell [49]. There were four heterozygous polymorphisms in the UMCC 855 *FRE1* sequence that lead to three missense mutations (Arg5His, Arg472Cys, Gly489Asp) and a gained stop in amino acid position 582 (S7 Table). With the only exception of mutation in amino acid position 5, that displayed a homozygous reference alleles, the alternative alleles were fixed in the evolved strain. Of particular significance was the gained stop that falls in the ferric reductase NAD binding domain, presumably resulting in a non-functional protein. The phenotype shown by the evolved strain suggests that, as occurs with copper, a non-functional Fre1p could protect against molybdate by lowering the molybdate import into the cell. However, the suggested hypothesis requires further study to be confirmed.

Metal toxicity may be caused by impaired DNA repair, inhibition of enzyme function or oxidative stress that originates from toxic levels of reactive oxygen species (ROS) stimulated directly or indirectly by metals [50–52]. ROS attack and damage all cellular macromolecules, leading to protein oxidation, lipid peroxidation and DNA damage [14]. For this reason, proteins related to oxidative stress were considered as candidate genes in our analysis and they were most often represented (4 out of 9 genes). Among these, Grx6p has proven to be directly involved in the oxidative stress response [53,54]. Indeed, the enzyme activity of Grx6p, cis-golgi localized monothiol glutaredoxin, consists of deglutathionylating mixed disulfides between glutathione and protein thiols, releasing reduced glutathione. Protein glutathionylation is a reversible mechanism for protecting protein thiols against irreversible oxidative modifications [55]. The missense mutation detected in this protein (serine > threonine, S8 Table) in position 93 is far from the active domain and because of the close similarity between the two amino acids substitution, we hypothesize that it is unlikely to have a large effect.

Some housekeeping processes appear to play a significant role in hyperoxia resistance [56]. In our QTL regions we found genes involved in controlling the activity of general transcription factors, such as Med2p, a component of RNA polymerase II [57,58], and metabolic processes, such as Nth1p, a neutral trehalase [59]. In addition, Pst2p shows similarity to flavodoxin and is induced by oxidative stress [60]. The specific function carried out in response to oxidative stress by these genes is not clearly understood. However, their involvement in the response against oxidative stress was reported by several authors [56,61–63]. Two heterozygous polymorphisms occurred in the *MED2* parental sequence (inframe insertion Asn345_Asn347dup and missense mutation Pro222Ser) and two occurred in the *PST2* parental sequence (missense mutations Ile186Ser and Glu185Lys) (S8 Table). All of these were fixed in the evolved strain but the effects of these mutations are difficult to predict. The *NTH1* sequence of the parental and evolved strains did not show any differences.

### Chromosomal variation in the evolved strain

*Saccharomyces cerevisiae* wine yeasts are known to display chromosomal copy number variation (polyploidy, aneuploidy) and rearranged chromosomes [64–66]. In this work, we observed that the median read depth of UMCC 2581 chromosome 1 was 1.5-fold greater than the median of the parental strain. A 3:2 ratio is indicative of an extra genomic copy in a diploid strain. A likely consequence of this aneuploidy was the higher than average expression of genes on chromosome 1 (Fig 4). Among the 117 verified ORF belonging to chromosome 1, we found two genes involved in GSH production (*CYS3*, *GDH3*), one gene involved in response to oxidative stress (*NTG1*) and four genes potentially relevant to metal resistance (*ERV46*, *VPS8*, *CCR4*, *DRS2*). *CYS3* and *GDH3* are both related to the synthesis of GSH precursor amino acids. In particular, *CYS3* encodes for the cystathionine γ-lyase, involved in the second step of the transsulfuration pathway, that yields cysteine from cystathionine [67]. *GDH3* encodes a NADP^+^-glutamate dehydrogenase that catalyzes the synthesis of glutamate from ammonia and alpha-ketoglutarate [68]. Interestingly, compared to *GLT1*, *GDH3* provides an alternative pathway for the synthesis of glutamate.

On chromosome 1 only one gene, *NTG1*, has a role in response to oxidative stress. The corresponding gene product, a DNA N-glycosylase and apurinic/apyrimidinic (AP) lyase, maintains genomic stability by removing a variety of oxidized pyrimidines from DNA in response to nuclear and mitochondrial oxidative stress [69,70].

There are no genes directly implicated in metal resistance mechanisms on chromosome 1. Nevertheless, the involvement of housekeeping genes *ERV46*, *VPS8*, *CCR4* and *DRS2* in metal resistance has been reported [49,71,72]. *ERV46*, a component of COPII vesicles [73], *VPS8*, a subunit of the CORVET complex [74] and *DRS2*, an aminophospholipid translocase [75], are all involved in vesicle-mediated transport processes. *CCR4* is a component of the CCR4-NOT transcriptional regulatory complex, able to affect gene expression both positively and negatively [76]. Overexpression of these genes could play a general, less direct role in promoting cell survival under stress conditions [50], and could thus positively affect the metal tolerance of UMCC 2581.

### Gene expression changes in the evolved strain

**Fig 7:**
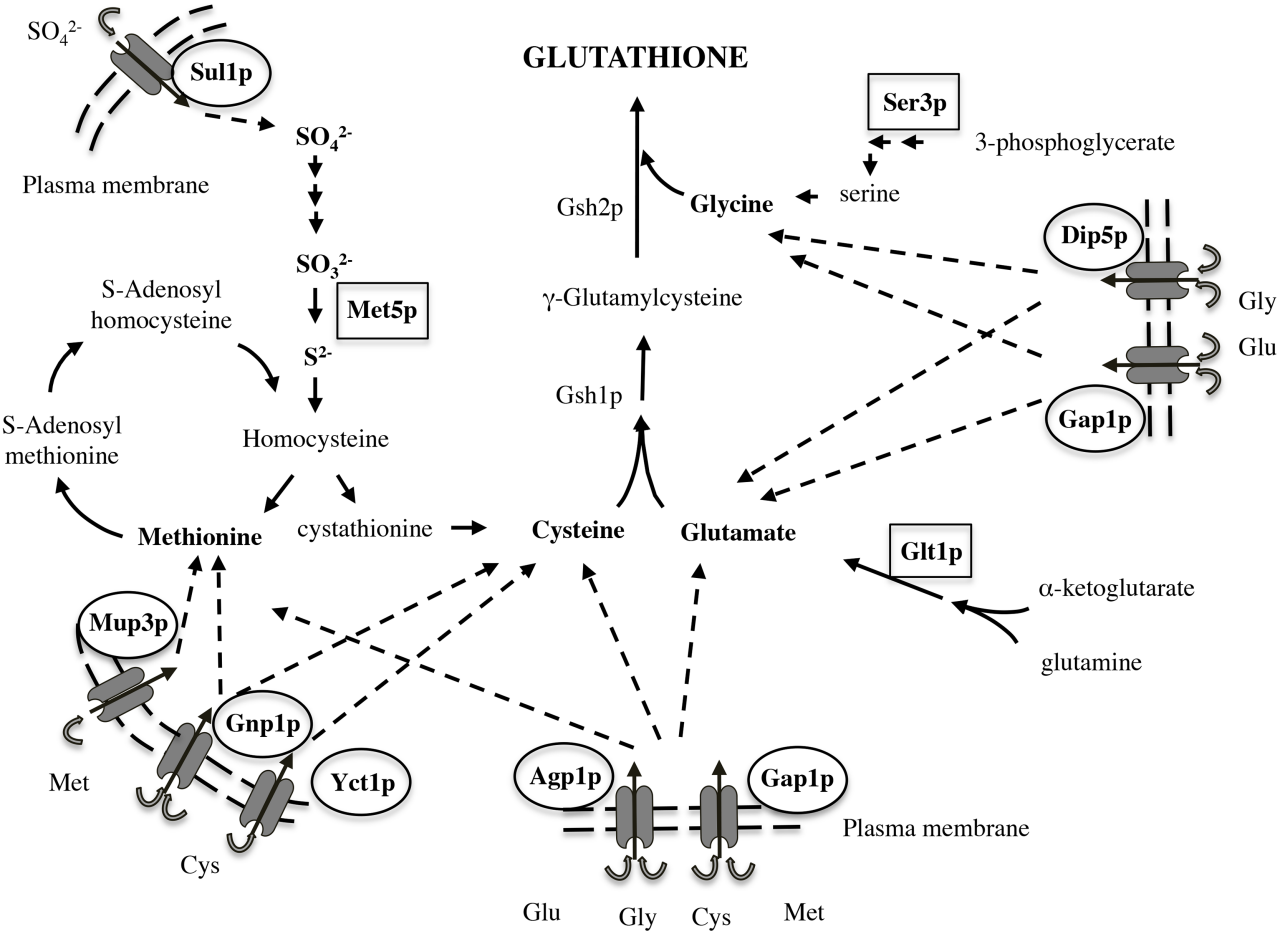
Glutathione precursor amino acids, permeases and enzymes, involved in the hypothesized mechanism for high GSH production. Methionine (Met) and cysteine (Cys) are transported by the permeases Mup3p, Gnp1p, Yct1p, and Agp1p. Glutamate (Glu) and glycine (Gly) are transported by Dip5p and Agp1p. The same amino acids are also transported by the common Gap1p permease. Sul1p: sulfate transporter; Met5p: sulfite reductase; Glt1p: glutamate synthetase; Ser3p: phosphoglycerate dehydrogenase; Gsh1p: γ-glutamylcysteine synthetase; Gsh2p: glutathione synthetase.

The comparison of the evolved and parental strains' gene expression provides some insight into molybdate resistance and GSH production. From genes up-regulated in UMCC 2581, we found enrichment of GO terms involved in transport activity and more specifically amino acid transporters (Fig 6). Analysis of the genes annotated with amino acid transmembrane transporter activity revealed that 7 out of 9 were genes are related to GSH precursor amino acids. Cysteine and methionine are transported by Yct1p, Mup3p, Gnp1p and Agp1p, glutamate and glycine by Dip5p (the gene with the largest expression difference) and Agp1, and all amino acids are transported by Gap1p, a general amino acid permease [77–79]. Moreover, other genes potentially related to GSH production were found among the up-regulated genes (S3 Table). In particular *SUL1*, encodes a high affinity sulfate permease [80]. *SUL1* along with *MET5*, which encodes for the β-subunit of the *S*. *cerevisiae* sulfite reductase [81], are involved in the sulfate assimilation pathway that precedes the synthesis of sulfur-containing amino acids cysteine and methionine [82]. *GLT1* encodes GOGAT (glutamate synthase) and synthesizes two molecules of glutamate out of one molecule of glutamine and one molecule of α-ketoglutarate [83]. Finally, Ser3p, a phosphoglycerate dehydrogenase, catalyzes the first reaction of serine and glycine biosynthesis from the glycolytic metabolite 3-phosphoglycerate [84]. Therefore, our results indicate that all the GSH precursor amino acids (sulfur-containing amino acids, glutamate and glycine) have genes that are up-regulated in their biosynthetic pathways (Fig 7). We interpret this data as evidence for an increase in transport of precursor amino acids from the media combined with increased production of glutathione. The gene expression patterns that we observe are of particular importance from the technological point of view: although gene regulation may differ in natural musts, the ability to gather precursor amino acids from the media for high GSH production is likely consistent across fermentations where musts differ year to year.

We found increased expression of two GSH-related genes involved in metal detoxification: *YCF1* and *ECM38*. The role of *YCF1* gene in providing resistance to heavy metals and xenobiotics as a GSH S-conjugate transporter was previously described [15]. The Y-glutamyltranspeptidase (Y-GT), encoded by *ECM38*, is the major GSH-degrading enzyme. Once GSH is transported into the vacuole, it is degraded by the vacuolar membrane-bound Y-GT and L-cysteinyl glycine dipeptidase by the cleavage of the Y-glutamyl moiety and the release of cysteinylglycine, which is further degraded to its constitutive amino acids [85]. A similar mechanism might be responsible for recycling of xenobiotics/metal-GSH complex stored in the vacuole, which can be excreted from cells [86,87] and provides a possible mechanism for molybdate resistance in the evolved strain.

## Conclusion

In this work, we identified genetic and transcriptional changes that may underlay the high GSH production demonstrated by the wine strain UMCC 2581. We identified two QTL and seven candidate genes along with 296 differentially expressed genes between parental and evolved strain. The combination of multiple QTL and expression changes suggest multiple factors underlying molybdate resistance and GSH production. A number of genes associated with more general transport pathways could also play a role in promoting cell survival under metal/oxidative stress conditions and in the GSH production and homeostasis. Further work will be needed to pinpoint the genes involved as well as examine the consequence of the chromosome 1 aneuploidy.

Our analysis of the transcriptional profiles revealed significant insight into the mechanism of the evolved strain’s phenotypes. Our main finding is the global over-expression of the amino acid permeases. Thus, the high GSH production phenotype is likely related to over-expression of amino acid permeases and precursor biosynthetic enzymes rather than alteration of the two GSH metabolic enzymes. GSH production and metabolism, transporter activity, vacuolar detoxification and oxidative stress response enzymes may also contribute to the molybdate resistance phenotype.

Regarding the application of molybdate as selective pressure to obtain evolved strains, this study provides an example of a combination of an evolution-based strategy to successfully obtain a yeast strain with a desired phenotype and genomic analysis to characterize the evolved strain.

## Supporting information

S1 Fig MCs selected for the sequenced cluster Resistant-Parental and Resistant-Evolved. Strain UMCC 855 was used to generate the monosporic clones (MCs) throughout this study.
(TIF)

S1 File Perl script used in analysis. (TXT)

S1 Table Summary of genome and transcriptome sequencing data generated in this study. Statistics from the Ion Torrent sequencing run.
(PDF)

S2 Table Synthetic must composition according to Giudici and Kunkee [27]. (PDF)

S3 Table UMCC 2581 compared to UMCC 855 as determined by RNA-seq. Genes with a significant expression change (FDR < 0.05) are shown, excluding those on chromosome 1 because of aneuploidy.
(PDF)

S4 Table Gene Ontology—Process. Comparison between gene expression levels of the UMCC 2581 and UMCC 855 strains. Gene Ontology (GO) process enriched for up-regulated and down-regulated genes in UMCC 2581 are reported.
(PDF)

S5 Table Gene Ontology—Function. Comparison between gene expression levels of the UMCC 2581 and UMCC 855 strains. Gene Ontology (GO) function enriched for up-regulated and down-regulated genes in UMCC 2581 are reported.
(PDF)

S6 Table Gene Ontology—Component. Comparison between gene expression levels of the UMCC 2581 and UMCC 855 strains. Gene Ontology (GO) component enriched for up-regulated and down-regulated genes in UMCC 2581 are reported.
(PDF)

S7 Table Genome locations and significant genomic variation of genes found in QTL peak in chromosome 12. Ref. Allele, reference allele as in S288c reference sequence. SNPs or InDels are considered heterozygous with the calculated allele frequency between 0.25–0.75 and coverage greater than 20. Predicted effect describes the variant using HGVS notation (http://www.hgvs.org/mutnomen/). Genes potentially relevant for the resistant phenotype are indicated in bold.
(PDF)

S8 Table Genome locations and significant genomic variation of genes found in QTL peak in chromosome 4. Ref. Allele, reference allele as in S288c reference sequence. SNPs or InDels are considered heterozygous with the calculated allele frequency between 0.25–0.75 and coverage greater than 20. Predicted effect describes the variant using HGVS notation (http://www.hgvs.org/mutnomen/). Genes potentially relevant for the resistant phenotype are indicated in bold.
(PDF)
