# Supplementary figures and images for: Genetic variation and expression changes associated with molybdate resistance from a glutathione producing wine strain of Saccharomyces cerevisiae
Source: PLoS One. 2017 Jul 6;12(7):e0180814. doi: 10.1371/journal.pone.0180814 (PMC5500363; doi:10.1371/journal.pone.0180814)

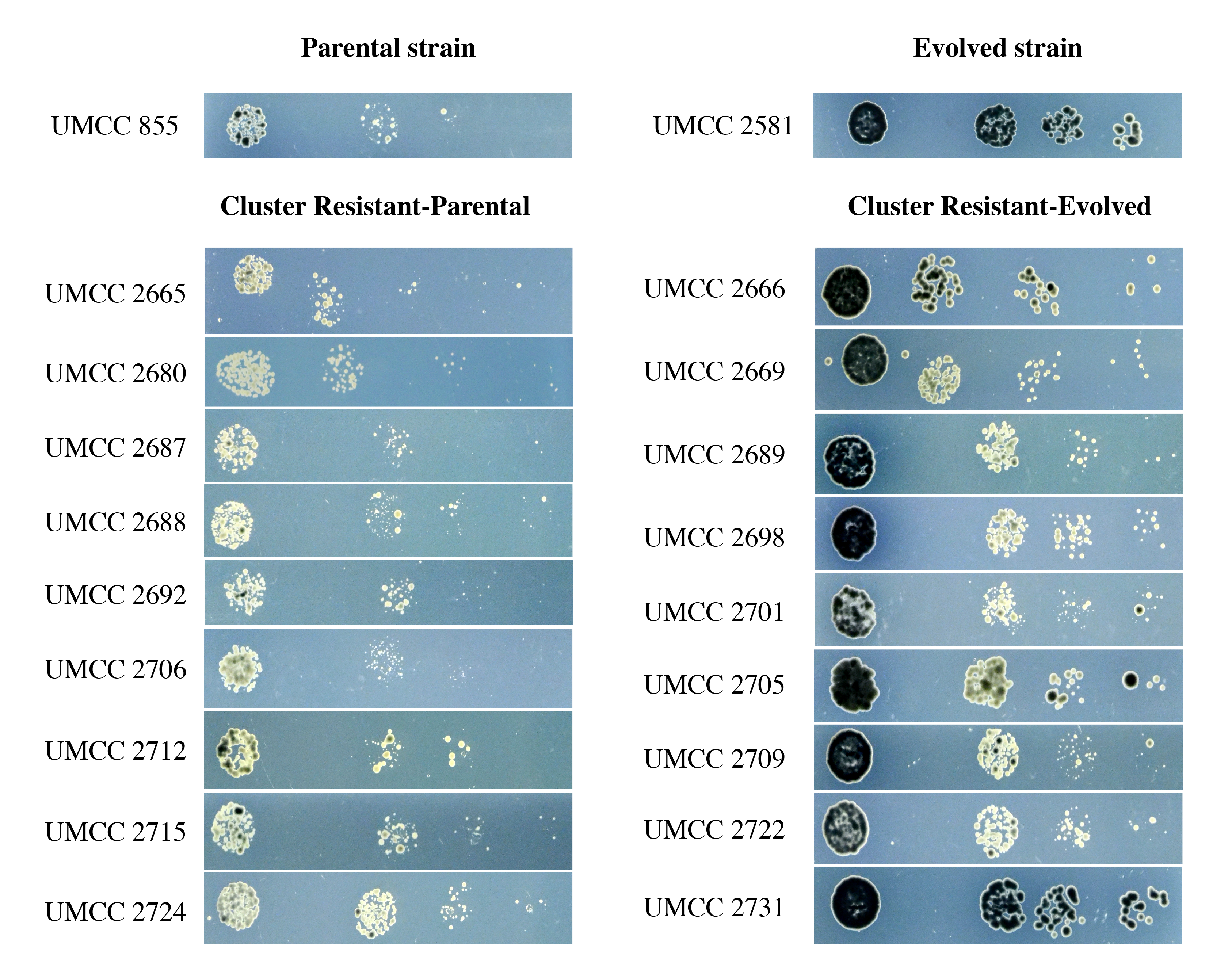

Supplement: S1 Fig — Strain UMCC 855 was used to generate the monosporic clones (MCs) throughout this study. (TIF) [file pone.0180814.s001.tif]
