# Supplementary material for: Genetic variation and expression changes associated with molybdate resistance from a glutathione producing wine strain of Saccharomyces cerevisiae
Source: PLoS One. 2017 Jul 6;12(7):e0180814. doi: 10.1371/journal.pone.0180814 (PMC5500363; doi:10.1371/journal.pone.0180814)
Supplement: S1 Table — Statistics from the Ion Torrent sequencing run. (PDF) [file pone.0180814.s003.pdf]

S1 Table. Summary of genome and transcriptome sequencing data generated in this study.
Statistics from the Ion Torrent sequencing run.

Sample	Bases	>=Q20 bases	Reads	Mean read length
UMCC 855 genome	1,668,850,209	1,411,272,981	11,632,368	143 bp
UMCC 2581 genome	1,389,700,415	1,195,000,705	9,860,882	141 bp
Cluster Resistant-Parental	975,820,078	808,919,415	6,507,162	150 bp
Cluster Resistant-Evolved	1,299,797,429	1,089,163,300	8,834,774	147 bp
Cluster Sensitive	820,028,716	695,173,250	5,751,073	143 bp
UMCC 855 transcriptome A	1,724,236,791	1,406,533,916	13,773,187	125 bp
UMCC 855 transcriptome B	1,432,513,561	1,171,599,932	11,254,086	127 bp
UMCC 855 transcriptome C	1,389,198,937	1,123,312,718	10,490,237	132 bp
UMCC 2581 transcriptome	1,237,629,410	1,005,812,569	9,742,043	127 bp
