# Supplementary material for: Genetic variation and expression changes associated with molybdate resistance from a glutathione producing wine strain of Saccharomyces cerevisiae
Source: PLoS One. 2017 Jul 6;12(7):e0180814. doi: 10.1371/journal.pone.0180814 (PMC5500363; doi:10.1371/journal.pone.0180814)
Supplement: S2 Table — (PDF) [file pone.0180814.s004.pdf]

S2 Table. Synthetic must composition according to Giudici and Kunkee [27].

Component	g/L
D-Fructose	110
D-Glucose	100
L-Malic Acid	3
Citric Acid	3
YNB (w/o AA & Amm.Sol.)	1.7
Tween 80	1E-03
Ergosterol	10E-03
Ammonium Sulfate	5

The media was titrated to pH 3.5 with KOH
