# Supplementary material for: Genetic variation and expression changes associated with molybdate resistance from a glutathione producing wine strain of Saccharomyces cerevisiae
Source: PLoS One. 2017 Jul 6;12(7):e0180814. doi: 10.1371/journal.pone.0180814 (PMC5500363; doi:10.1371/journal.pone.0180814)
Supplement: S3 Table — Genes with a significant expression change (FDR < 0.05) are shown, excluding those on chromosome 1 because of aneuploidy. (PDF) [file pone.0180814.s005.pdf]

S3 Table. UMCC 2581 compared to UMCC 855 as determined by RNA-seq.
Genes with a significant expression change (FDR < 0.05) are shown, excluding those on chromosome 1 because of aneuploidy.

Systematic gene name	Standard gene name	Gene description	Fold change	p-adjusted
YPL265W	*DIP5*	Dicarboxylic amino acid permease	20.44	7.31E-15
YDR536W	*STL1*	Glycerol proton symporter of the plasma membrane	18.73	1.40E-06
YMR011W	*HXT2*	High-affinity glucose transporter of the major facilitator superfamily	16.20	6.06E-14
YHR033W		Putative protein of unknown function	12.00	1.76E-25
YHR096C	*HXT5*	Hexose transporter with moderate affinity for glucose	10.94	9.69E-06
YLR413W	*INA1*	Putative protein of unknown function	10.73	7.89E-27
YDR508C	*GNP1*	High-affinity glutamine permease	10.45	1.93E-12
YFL053W	*DAK2*	Dihydroxyacetone kinase	9.64	4.13E-02
YKL187C	*FAT3*	Protein required for fatty acid uptake	8.91	1.68E-02
YGL089C	*MF(ALPHA)2*	Mating pheromone alpha-factor, made by alpha cells	8.50	4.51E-02
YBR296C	*PHO89*	Plasma membrane Na+/Pi cotransporter	8.17	1.55E-02
YOL013W-A		Putative protein of unknown function	7.59	2.23E-04
YEL065W	*SIT1*	Ferrioxamine B transporter	6.75	1.76E-25
YNL194C		Integral membrane protein	6.40	2.40E-06
YBR294W	*SUL1*	High affinity sulfate permease of the SulP anion transporter family	6.31	4.94E-14
YDL222C	*FMP45*	Integral membrane protein localized to mitochondria	6.12	3.99E-14
YBR117C	*TKL2*	Transketolase	6.02	1.00E-04
YLR265C	*NEJ1*	Protein involved in regulation of nonhomologous end joining	5.97	9.33E-04
YDL241W		Putative protein of unknown function	5.34	1.73E-02
YOR028C	*CIN5*	Basic leucine zipper (bZIP) transcription factor of the yAP-1 family	4.85	4.98E-07
YPL223C	*GRE1*	Hydrophilin essential in desiccation-rehydration process	4.82	5.07E-15
YKR093W	*PTR2*	Integral membrane peptide transporter	4.49	3.17E-03
YDR342C	*HXT7*	High-affinity glucose transporter	4.28	4.89E-02
YFR032C	*RRT5*	Putative protein of unknown function	4.27	9.73E-03
YOL052C-A	*DDR2*	Multi-stress response protein	4.21	6.83E-07
YNL195C		Protein of unknown function	4.00	9.78E-09
YNL145W	*MFA2*	Mating pheromone a-factor	3.49	1.73E-02
YJL089W	*SIP4*	C6 zinc cluster transcriptional activator	3.29	3.41E-03
YEL039C	*CYC7*	Cytochrome c isoform 2, expressed under hypoxic conditions	3.12	1.71E-02
YMR085W		Putative protein of unknown function	3.09	2.35E-06
YGR043C	*NQM1*	Transaldolase of unknown function	3.07	1.48E-03
YHR022C		Putative protein of unknown function	3.05	1.22E-02

YDL039C	*PRM7*	Pheromone-regulated protein	3.05	1.49E-05
YNR014W		Putative protein of unknown function	3.03	1.34E-06
YPR002W	*PDH1*	Putative 2-methylcitrate dehydratase	3.03	2.27E-17
YHL026C		Putative protein of unknown function	2.97	2.61E-05
YDR534C	*FIT1*	Mannoprotein that is incorporated into the cell wall	2.86	3.52E-03
YOR161C	*PNS1*	Protein of unknown function	2.85	3.99E-07
YML116W	*ATR1*	Multidrug efflux pump of the major facilitator superfamily	2.72	3.55E-21
YIL121W	*QDR2*	Plasma membrane transporter of the major facilitator superfamily	2.68	8.79E-13
YLR174W	*IDP2*	Cytosolic NADP-specific isocitrate dehydrogenase	2.68	3.57E-09
YOL047C	*LDS2*	Protein Involved in spore wall assembly	2.65	2.44E-02
YNL279W	*PRM1*	Pheromone-regulated multispanning membrane protein	2.57	5.41E-04
YLL061W	*MMP1*	High-affinity S-methylmethionine permease	2.54	2.61E-05
YGR248W	*SOL4*	6-phosphogluconolactonase	2.51	5.32E-03
YDL214C	*PRR2*	Serine/threonine protein kinase	2.48	1.80E-02
YGR121C	*MEP1*	Ammonium permease	2.38	1.22E-09
YDL223C	*HBT1*	Shmoo tip protein, substrate of Hub1p ubiquitin-like protein	2.37	2.23E-04
YOL158C	*ENB1*	Endosomal ferric enterobactin transporter	2.34	8.98E-08
YOL155C	*HPF1*	Haze-protective mannoprotein	2.34	2.90E-09
YBR244W	*GPX2*	Phospholipid hydroperoxide glutathione peroxidase	2.34	6.51E-04
YDL049C	*KNH1*	Protein with similarity to Kre9p	2.32	1.09E-04
YBR008C	*FLR1*	Plasma membrane transporter of the major facilitator superfamily	2.29	2.40E-11
YCR021C	*HSP30*	Negative regulator of the H(+)-ATPase Pma1p	2.22	2.06E-07
YLL057C	*JLP1*	Fe(II)-dependent sulfonate/alpha-ketoglutarate dioxygenase	2.20	1.19E-02
YJL108C	*PRM10*	Pheromone-regulated protein	2.19	1.41E-09
YGR197C	*SNG1*	Protein involved in resistance to nitrosoguanidine and 6-azauracil	2.19	2.09E-02
YOL020W	*TAT2*	High affinity tryptophan and tyrosine permease	2.19	2.48E-03
YDR011W	*SNQ2*	Plasma membrane ATP-binding cassette (ABC) transporter	2.16	2.61E-05
YJL191W	*RPS14B*	Protein component of the small (40S) ribosomal subunit	2.15	1.21E-06
YIR032C	*DAL3*	Ureidoglycolate lyase	2.13	1.57E-03
YHL016C	*DUR3*	Plasma membrane transporter for both urea and polyamines	2.12	6.87E-03
YNL058C		Putative protein of unknown function	2.09	2.61E-03
YFL026W	*STE2*	Receptor for alpha-factor pheromone	2.05	6.33E-03
YCL025C	*AGP1*	Low-affinity amino acid permease with broad substrate range	2.05	1.24E-04
YDR216W	*ADR1*	Carbon source-responsive zinc-finger transcription factor	2.02	1.61E-02
YLL055W	*YCT1*	High-affinity cysteine-specific transporter	2.00	1.65E-04
YML091C	*RPM2*	Protein subunit of mitochondrial RNase P	1.98	3.73E-07
YJL107C		Putative protein of unknown function	1.98	1.26E-02
YMR169C	*ALD3*	Cytoplasmic aldehyde dehydrogenase	1.98	7.98E-05

YIR031C	*DAL7*	Malate synthase	1.97	1.58E-04
YOR374W	*ALD4*	Mitochondrial aldehyde dehydrogenase	1.96	7.12E-05
YKL161C	*KDX1*	Protein kinase	1.94	6.85E-08
YDL171C	*GLT1*	NAD(+)-dependent glutamate synthase (GOGAT)	1.94	6.35E-08
YIL101C	*XBP1*	Transcriptional repressor	1.93	3.08E-02
YGR243W	*MPC3*	Highly conserved subunit of mitochondrial pyruvate carrier	1.93	7.25E-03
YOR346W	*REV1*	Deoxycytidyl transferase	1.92	4.94E-03
YDR259C	*YAP6*	Basic leucine zipper (bZIP) transcription factor	1.91	6.87E-03
YOR356W	*CIR2*	Putative ortholog of human ETF-dH	1.91	5.73E-05
YEL040W	*UTR2*	Chitin transglycosylase	1.91	2.84E-06
YMR058W	*FET3*	Ferro-O2-oxidoreductase	1.90	1.83E-06
YHR023W	*MYO1*	Type II myosin heavy chain	1.87	3.11E-03
YIR027C	*DAL1*	Allantoinase	1.85	2.73E-02
YBR021W	*FUR4*	Plasma membrane localized uracil permease	1.85	2.31E-02
YGR052W	*FMP48*	Putative protein of unknown function	1.83	3.36E-06
YMR032W	*HOF1*	Protein that regulates actin cytoskeleton organization	1.83	3.92E-02
YNR044W	*AGA1*	Anchorage subunit of a-agglutinin of a-cells	1.82	3.24E-02
YLR297W		Protein of unknown function	1.81	1.19E-02
YOL084W	*PHM7*	Protein of unknown function	1.80	3.44E-05
YLR304C	*ACO1*	Aconitase	1.79	6.05E-10
YPR149W	*NCE102*	Protein of unknown function	1.79	8.67E-05
YIL024C		Putative protein of unknown function	1.79	4.51E-02
YGR088W	*CTT1*	Cytosolic catalase T	1.77	1.46E-02
YJR124C		Putative protein of unknown function	1.75	5.32E-03
YBR150C	*TBS1*	Putative protein of unknown function	1.74	1.73E-02
YOL126C	*MDH2*	Cytoplasmic malate dehydrogenase	1.73	1.07E-03
YNL129W	*NRK1*	Nicotinamide riboside kinase	1.72	3.59E-03
YMR246W	*FAA4*	Long chain fatty acyl-CoA synthetase	1.68	1.34E-05
YBR293W	*VBA2*	Permease of basic amino acids in the vacuolar membrane	1.68	2.44E-02
YML076C	*WAR1*	Homodimeric Zn2Cys6 zinc finger transcription factor	1.65	3.05E-03
YPL017C	*IRC15*	Microtubule associated protein	1.65	8.88E-03
YER024W	*YAT2*	Carnitine acetyltransferase	1.63	1.08E-02
YLR108C		Protein of unknown function	1.61	3.88E-04
YJL172W	*CPS1*	Vacuolar carboxypeptidase S	1.60	1.61E-04
YHR097C		Putative protein of unknown function	1.60	2.01E-02
YBR005W	*RCR1*	Protein of the ER membrane involved in cell wall chitin deposition	1.60	1.25E-02
YIR029W	*DAL2*	Allantoicase	1.60	3.82E-02
YLR346C		Putative protein of unknown function found in mitochondria	1.59	4.58E-02
YJL078C	*PRY3*	Cell wall-associated protein involved in export of acetylated sterols	1.59	4.99E-03
YGR023W	*MTL1*	Putative plasma membrane sensor	1.59	3.60E-02
YER081W	*SER3*	3-phosphoglycerate dehydrogenase	1.59	3.31E-05
YDL025C	*RTK1*	Putative protein kinase, potentially phosphorylated by Cdc28p	1.59	4.45E-04

YGL114W		Putative protein of unknown function	1.58	3.24E-03
YPR160W	*GPH1*	Glycogen phosphorylase required for the mobilization of glycogen	1.58	6.87E-03
YIL155C	*GUT2*	Mitochondrial glycerol-3-phosphate dehydrogenase	1.57	4.51E-02
YOR382W	*FIT2*	Mannoprotein that is incorporated into the cell wall	1.57	1.73E-02
YKR039W	*GAP1*	General amino acid permease	1.57	4.94E-02
YLR401C	*DUS3*	Dihydrouridine synthase	1.56	3.79E-02
YJL035C	*TAD2*	Subunit of tRNA-specific adenosine-34 deaminase	1.56	4.06E-02
YLL027W	*ISA1*	Protein required for maturation of mitochondrial [4Fe-4S] proteins	1.55	1.70E-02
YNL068C	*FKH2*	Forkhead family transcription factor	1.55	4.80E-02
YKL051W	*SFK1*	Plasma membrane protein that may act to generate normal levels of PI4P	1.55	4.80E-02
YGR092W	*DBF2*	Ser/Thr kinase involved in transcription and stress response	1.55	2.54E-02
YBR183W	*YPC1*	Alkaline ceramidase	1.55	4.56E-02
YDR334W	*SWR1*	Swi2/Snf2-related ATPase	1.54	1.69E-02
YGR238C	*KEL2*	Protein that negatively regulates mitotic exit	1.54	3.12E-02
YLR299W	*ECM38*	Gamma-glutamyltranspeptidase	1.54	1.71E-03
YPL014W		Putative protein of unknown function	1.53	1.88E-02
YGR239C	*PEX21*	Peroxin required for peroxisomal matrix protein targeting	1.53	1.36E-02
YLR168C	*UPS2*	Mitochondrial intermembrane space protein	1.53	3.03E-03
YBR291C	*CTP1*	Mitochondrial inner membrane citrate transporter	1.53	2.73E-02
YHR146W	*CRP1*	Protein that binds to cruciform DNA structures	1.52	6.25E-03
YDR034C	*LYS14*	Transcriptional activator involved in regulating lysine biosynthesis	1.52	1.51E-03
YMR189W	*GCV2*	P subunit of the mitochondrial glycine decarboxylase complex	1.51	3.24E-03
YNL074C	*MLF3*	Serine-rich protein of unknown function	1.49	9.65E-03
YNL311C	*SKP2*	F-box protein of unknown function	1.49	4.51E-02
YLR414C	*PUN1*	Plasma membrane protein with a role in cell wall integrity	1.48	5.57E-03
YJL088W	*ARG3*	Ornithine carbamoyltransferase	1.47	6.69E-03
YGR090W	*UTP22*	Component of the small-subunit processome	1.47	4.33E-02
YMR318C	*ADH6*	NADPH-dependent medium chain alcohol dehydrogenase	1.46	4.46E-03
YMR300C	*ADE4*	Phosphoribosylpyrophosphate amidotransferase (PRPPAT)	1.46	2.61E-03
YHL036W	*MUP3*	Low affinity methionine permease	1.45	4.51E-02
YOR120W	*GCY1*	Glycerol dehydrogenase	1.45	2.93E-02
YHR216W	*IMD2*	Inosine monophosphate dehydrogenase	1.43	1.46E-02
YMR016C	*SOK2*	Nuclear protein that negatively regulates pseudohyphal differentiation	1.43	4.13E-02
YER164W	*CHD1*	Chromatin remodeler that regulates various aspects of transcription	1.42	4.51E-02
YER056C	*FCY2*	Purine-cytosine permease	1.41	1.80E-02
YOR136W	*IDH2*	Subunit of mitochondrial NAD(+)-dependent isocitrate dehydrogenase	1.40	1.17E-02

YNL037C	*IDH1*	Subunit of mitochondrial NAD(+)-dependent isocitrate dehydrogenase	1.39	1.48E-02
YJL200C	*ACO2*	Putative mitochondrial aconitase isozyme	1.38	9.84E-03
YJL141C	*YAK1*	Serine-threonine protein kinase	1.38	4.86E-02
YMR070W	*MOT3*	Transcriptional repressor and activator with two C2-H2 zinc fingers	1.37	4.51E-02
YGR061C	*ADE6*	Formylglycinamidine-ribonucleotide (FGAM)-synthetase	1.37	1.46E-02
YNR016C	*ACC1*	Acetyl-CoA carboxylase, biotin containing enzyme	1.36	5.09E-03
YER069W	*ARG5,6*	Acetylglutamate kinase and N-acetyl-gamma-glutamyl-phosphate reductase	1.36	1.73E-02
YDL140C	*RPO21*	RNA polymerase II largest subunit B220	1.36	1.80E-02
YDR135C	*YCF1*	Vacuolar glutathione S-conjugate transporter	1.36	3.08E-02
YGL009C	*LEU1*	Isopropylmalate isomerase	1.35	1.33E-02
YGL077C	*HNM1*	Plasma membrane transporter for choline, ethanolamine, and carnitine	1.35	3.79E-02
YDR074W	*TPS2*	Phosphatase subunit of the trehalose-6-P synthase/phosphatase complex	1.35	1.66E-02
YJR137C	*MET5*	Sulfite reductase beta subunit	1.31	4.00E-02
YBR214W	*SDS24*	Protein involved in cell separation during budding	0.75	4.85E-02
YNL160W	*YGP1*	Cell wall-related secretory glycoprotein	0.74	4.80E-02
YER152C		Protein with 2-aminoadipate transaminase activity	0.72	3.44E-02
YDL048C	*STP4*	Protein containing a Kruppel-type zinc-finger domain	0.71	3.16E-02
YJL056C	*ZAP1*	Zinc-regulated transcription factor	0.71	2.73E-02
YOR188W	*MSB1*	Protein of unknown function	0.71	4.51E-02
YER088C	*DOT6*	Protein involved in rRNA and ribosome biogenesis	0.70	4.48E-03
YLR136C	*TIS11*	mRNA-binding protein expressed during iron starvation	0.70	4.51E-02
YHR188C	*GPI16*	Subunit of the glycosylphosphatidylinositol transamidase complex	0.70	1.28E-02
YNL200C		NADHX epimerase	0.70	4.80E-02
YCR098C	*GIT1*	Plasma membrane permease	0.69	4.28E-02
YFL042C	*LAM5*	Putative sterol transfer protein	0.69	4.50E-02
YGL156W	*AMS1*	Vacuolar alpha mannosidase	0.68	3.21E-03
YOR344C	*TYE7*	Serine-rich protein that contains a bHLH DNA binding motif	0.68	1.73E-02
YNR039C	*ZRG17*	Endoplasmic reticulum zinc transporter	0.68	1.01E-02
YBR169C	*SSE2*	Member of the heat shock protein 70 (HSP70) family	0.68	1.14E-02
YBR201W	*DER1*	ER membrane protein that promotes export of misfolded polypeptides	0.68	4.80E-02
YPL026C	*SKS1*	Putative serine/threonine protein kinase	0.68	4.91E-02
YJL082W	*IML2*	Protein of unknown function	0.67	5.54E-03
YOR321W	*PMT3*	Protein O-mannosyltransferase	0.67	5.77E-03
YJL020C	*BBC1*	Protein possibly involved in assembly of actin patches	0.67	2.50E-03
YLR045C	*STU2*	Microtubule-associated protein (MAP) of the XMAP215/Dis1 family	0.66	2.40E-02

YKL029C	*MAE1*	Mitochondrial malic enzyme	0.66	3.24E-03
YOL007C	*CSI2*	Protein of unknown function	0.66	2.12E-02
YNR026C	*SEC12*	Guanine nucleotide exchange factor (GEF)	0.66	2.51E-02
YNL289W	*PCL1*	Cyclin, interacts with cyclin-dependent kinase Pho85p	0.66	6.69E-03
YOL055C	*THI20*	Trifunctional enzyme of thiamine biosynthesis, degradation and salvage	0.65	7.80E-04
YJR061W		Putative protein of unknown function	0.65	3.05E-03
YPR091C	*NVJ2*	Lipid-binding ER protein, enriched at nucleus-vacuolar junctions (NVJ)	0.65	4.44E-02
YNL168C	*FMP41*	Putative protein of unknown function	0.64	2.42E-02
YIR035C		Putative cytoplasmic short-chain dehydrogenase/reductase	0.64	7.93E-03
YNR040W		Putative protein of unknown function	0.64	4.85E-02
YDR320C	*SWA2*	Auxilin-like protein involved in vesicular transport	0.64	1.76E-03
YOR274W	*MOD5*	Delta 2-isopentenyl pyrophosphate:tRNA isopentenyl transferase	0.64	4.32E-03
YIL124W	*AYR1*	Bifunctional triacylglycerol lipase and 1-acyl DHAP reductase	0.63	1.14E-02
YPL134C	*ODC1*	Mitochondrial inner membrane transporter	0.63	2.55E-02
YER175C	*TMT1*	Trans-aconitate methyltransferase	0.63	3.34E-02
YGL256W	*ADH4*	Alcohol dehydrogenase isoenzyme type IV	0.63	1.25E-02
YOL082W	*ATG19*	Receptor protein for the cytoplasm-to-vacuole targeting (Cvt) pathway	0.63	3.01E-03
YLR126C		Putative glutamine amidotransferase	0.63	4.81E-03
YKR003W	*OSH6*	Member of an oxysterol-binding protein family	0.62	1.43E-02
YCL028W	*RNQ1*	[PIN(+)] prion	0.62	1.51E-03
YPL277C		Putative protein of unknown function	0.62	1.44E-03
YOL083W	*ATG34*	Receptor protein involved in selective autophagy during starvation	0.61	4.86E-02
YMR170C	*ALD2*	Cytoplasmic aldehyde dehydrogenase	0.61	2.52E-02
YIL119C	*RPI1*	Transcription factor, allelic differences between S288C and Sigma1278b	0.60	4.45E-02
YOR003W	*YSP3*	Putative precursor to the subtilisin-like protease III	0.60	4.35E-02
YER158C		Protein of unknown function	0.60	3.05E-03
YLR059C	*REX2*	3'-5' RNA exonuclease	0.59	1.25E-02
YPR196W		Putative maltose-responsive transcription factor	0.59	4.42E-04
YBR045C	*GIP1*	Meiosis-specific regulatory subunit of the Glc7p protein phosphatase	0.59	4.32E-03
YDR205W	*MSC2*	Endoplasmic reticulum zinc transporter	0.59	4.53E-03
YOR125C	*CAT5*	Protein required for ubiquinone (Coenzyme Q) biosynthesis	0.58	1.70E-02
YMR096W	*SNZ1*	Protein involved in vitamin B6 biosynthesis	0.58	1.80E-04
YDR400W	*URH1*	Uridine nucleosidase (uridine-cytidine N-ribohydrolase)	0.58	3.51E-04
YCL049C		Protein of unknown function	0.57	2.01E-04
YJL157C	*FAR1*	CDK inhibitor and nuclear anchor	0.56	1.77E-04
YGR144W	*THI4*	Thiazole synthase	0.56	3.01E-03
YOL128C	*YGK3*	Protein kinase related to mammalian GSK-3 glycogen synthase kinases	0.56	1.97E-03
YDR506C	*GMC1*	Protein involved in meiotic progression	0.55	3.79E-02

YER015W	*FAA2*	Medium chain fatty acyl-CoA synthetase	0.55	4.50E-06
YMR020W	*FMS1*	Polyamine oxidase	0.55	1.04E-02
YML054C	*CYB2*	Cytochrome b2 (L-lactate cytochrome-c oxidoreductase)	0.55	6.29E-03
YLR231C	*BNA5*	Kynureninase	0.54	5.13E-06
YKL049C	*CSE4*	Centromere protein that resembles histone H3	0.54	3.63E-02
YGL185C		Putative protein with sequence similar to hydroxyacid dehydrogenases	0.53	3.47E-03
YNR075W	*COS10*	Protein of unknown function	0.53	1.61E-04
YBL049W	*MOH1*	Protein of unknown function, essential for stationary phase survival	0.53	1.13E-02
YOR092W	*ECM3*	Non-essential protein of unknown function	0.52	7.10E-04
YJR025C	*BNA1*	3-hydroxyanthranilic acid dioxygenase	0.52	4.14E-04
YOR071C	*NRT1*	High-affinity nicotinamide riboside transporter	0.52	2.14E-02
YFR055W	*IRC7*	Beta-lyase involved in the production of thiols	0.51	3.72E-06
YGL121C	*GPG1*	Proposed gamma subunit of the heterotrimeric G protein	0.50	1.07E-03
YER130C	*COM2*	Transcription factor that binds IME1 Upstream Activation Signal (UAS)ru	0.50	3.35E-06
YLR237W	*THI7*	Plasma membrane transporter responsible for the uptake of thiamine	0.50	8.73E-10
YJR149W		Putative protein of unknown function	0.50	1.15E-02
YLR154C	*RNH203*	Ribonuclease H2 subunit	0.49	1.68E-02
YOR301W	*RAX1*	Protein involved in bud site selection during bipolar budding	0.48	1.61E-04
YFL050C	*ALR2*	Probable Mg(2+) transporter	0.48	9.41E-08
YGL257C	*MNT2*	Mannosyltransferase	0.47	8.65E-07
YDR379C-A	*SDH6*	Mitochondrial protein involved in assembly of succinate dehydrogenase	0.47	4.03E-02
YGL262W		Putative protein of unknown function	0.47	9.84E-03
YIL072W	*HOP1*	Meiosis-specific protein required for chromosome synapsis	0.47	3.17E-03
YLR004C	*THI73*	Putative plasma membrane permease	0.47	1.34E-09
YPL191C		Putative protein of unknown function	0.46	5.64E-03
YIL099W	*SGA1*	Intracellular sporulation-specific glucoamylase	0.46	8.08E-03
YDR461C-A		Putative protein of unknown function	0.46	2.72E-03
YDR503C	*LPP1*	Lipid phosphate phosphatase	0.45	1.44E-04
YNL254C	*RTC4*	Protein of unknown function	0.45	6.80E-07
YHL028W	*WSC4*	Endoplasmic reticulum (ER) membrane protein	0.45	1.80E-04
YHL048W	*COS8*	Nuclear membrane protein	0.45	4.92E-05
YPR006C	*ICL2*	2-methylisocitrate lyase of the mitochondrial matrix	0.44	2.24E-02
YMR095C	*SNO1*	Protein of unconfirmed function	0.44	9.73E-03
YMR199W	*CLN1*	G1 cyclin involved in regulation of the cell cycle	0.43	4.05E-10
YOR386W	*PHR1*	DNA photolyase involved in photoreactivation	0.43	3.96E-02
YDR243C	*PRP28*	RNA binding protein	0.43	3.03E-04
YPL248C	*GAL4*	DNA-binding transcription factor required for activating GAL genes	0.43	1.13E-04
YBL029W		Non-essential protein of unknown function	0.42	6.28E-08
YKL043W	*PHD1*	Transcriptional activator that enhances	0.42	4.98E-12

		pseudohyphal growth		
YGL205W	*POX1*	Fatty-acyl coenzyme A oxidase	0.41	2.86E-08
YOR049C	*RSB1*	Suppressor of sphingoid LCB sensitivity of an LCB-lyase mutation	0.41	4.98E-03
YHR140W		Putative integral membrane protein of unknown function	0.40	7.25E-03
YBR033W	*EDS1*	Putative zinc cluster protein, predicted to be a transcription factor	0.40	2.97E-02
YPL258C	*THI21*	Hydroxymethylpyrimidine (HMP) and HMP-phosphate kinase	0.39	1.21E-12
YBR072W	*HSP26*	Small heat shock protein (sHSP) with chaperone activity	0.38	3.06E-09
YDR406W	*PDR15*	Plasma membrane ATP binding cassette (ABC) transporter	0.38	4.47E-17
YJR079W		Putative protein of unknown function	0.38	5.69E-03
YKL132C	*RMA1*	Putative dihydrofolate synthetase	0.35	4.19E-05
YLR070C	*XYL2*	Xylitol dehydrogenase	0.34	4.34E-03
YBR240C	*THI2*	Transcriptional activator of thiamine biosynthetic genes	0.34	1.42E-14
YBL048W	*RRT1*	Protein of unknown function	0.34	1.83E-02
YNL065W	*AQR1*	Plasma membrane transporter of the major facilitator superfamily	0.32	1.80E-15
YMR182C	*RGM1*	Putative zinc finger DNA binding transcription factor	0.32	4.81E-02
YER060W-A	*FCY22*	Putative purine-cytosine permease	0.30	1.52E-02
YEL049W	*PAU2*	Member of the seripauperin multigene family	0.29	3.79E-02
YIL160C	*POT1*	3-ketoacyl-CoA thiolase with broad chain length specificity	0.29	7.72E-06
YPR121W	*THI22*	Protein with similarity to hydroxymethylpyrimidine phosphate kinases	0.29	1.99E-10
YJR156C	*THI11*	Protein involved in synthesis of the thiamine precursor HMP	0.28	3.53E-07
YIR039C	*YPS6*	Putative GPI-anchored aspartic protease	0.26	3.43E-04
YER060W	*FCY21*	Putative purine-cytosine permease	0.25	6.94E-11
YGR287C	*IMA1*	Major isomaltase (alpha-1,6-glucosidase/alpha-methylglucosidase)	0.24	9.68E-10
YFR026C	*ULI1*	Putative protein of unknown function	0.23	4.82E-07
YGL258W	*VEL1*	Protein of unknown function	0.18	5.41E-09
YOR161W-B		Protein of unknown function	0.15	3.24E-03
YOL131W		Putative protein of unknown function	0.15	3.17E-05
YNL012W	*SPO1*	Meiosis-specific prospore protein	0.15	7.30E-03
YDL244W	*THI13*	Protein involved in synthesis of the thiamine precursor HMP	0.14	2.51E-16
YDR380W	*ARO10*	Phenylpyruvate decarboxylase	0.12	1.80E-02
YOR387C		Putative protein of unknown function	0.09	6.50E-30
YMR319C	*FET4*	Low-affinity Fe(II) transporter of the plasma membrane	0.07	3.64E-32
YGR108W	*CLB1*	B-type cyclin involved in cell cycle progression	0	7.49E-22
YGR109C	*CLB6*	B-type cyclin involved in DNA replication during S phase	0	5.59E-03
YGR288W	*MAL13*	MAL-activator protein	0	8.99E-17

YGR289C	*MAL11*	High-affinity maltose transporter (alpha-glucoside transporter)	0	2.17E-06
YIR042C		Putative protein of unknown function	0	5.17E-14
