# Supplementary material for: Genetic variation and expression changes associated with molybdate resistance from a glutathione producing wine strain of Saccharomyces cerevisiae
Source: PLoS One. 2017 Jul 6;12(7):e0180814. doi: 10.1371/journal.pone.0180814 (PMC5500363; doi:10.1371/journal.pone.0180814)
Supplement: S4 Table — Comparison between gene expression levels of the UMCC 2581 and UMCC 855 strains. Gene Ontology (GO) process enriched for up-regulated and down-regulated genes in UMCC 2581 are reported. (PDF) [file pone.0180814.s006.pdf]

S4 Table. Gene Ontology - Process.
Comparison between gene expression levels of the UMCC 2581 and UMCC 855 strains. Gene Ontology (GO) process enriched for up-regulated and down-regulated genes in UMCC 2581 are reported.

Process Ontology

Gene Ontology Enrichment, up-regulated genes

Gene Ontology ID	Gene Ontology term	Cluster frequency	Background frequency	p-value	FDR	Expected False Positives	Genes annotated to the term
6820	anion transport	19 out of 140 genes, 13.6%	119 out of 4737 background genes, 2.5%	5.42E-07	0.00	0.00	*CTP1/ YBR291C, VBA2/ YBR293W, SUL1/ YBR294W, PHO89/ YBR296C, AGP1/ YCL025C, YCF1/ YDR135C, GNP1/ YDR508C, HNM1/ YGL077C, MPC3/ YGR243W, MUP3/ YHL036W, FAT3/ YKL187C, GAP1/ YKR039W, YCT1/ YLL055W, ATR1/ YML116W, FET3/ YMR058W, FAA4/ YMR246W, TAT2/ YOL020W, ENB1/ YOL158C, DIP5/ YPL265W*
46942	carboxylic acid transport	14 out of 140 genes, 10.0%	67 out of 4737 background genes, 1.4%	2.71E-06	0.00	0.00	*CTP1/ YBR291C, VBA2/ YBR293W, AGP1/ YCL025C, YCF1/ YDR135C, GNP1/ YDR508C, HNM1/ YGL077C, MPC3/ YGR243W, MUP3/ YHL036W, FAT3/ YKL187C, GAP1/ YKR039W, YCT1/ YLL055W, FAA4/ YMR246W, TAT2/ YOL020W, DIP5/ YPL265W*
15849	organic acid transport	14 out of 140 genes, 10.0%	68 out of 4737 background genes, 1.4%	3.33E-06	0.00	0.00	*CTP1/ YBR291C, VBA2/ YBR293W, AGP1/ YCL025C, YCF1/ YDR135C, GNP1/ YDR508C, HNM1/ YGL077C, MPC3/ YGR243W, MUP3/ YHL036W, FAT3/ YKL187C, GAP1/ YKR039W, YCT1/ YLL055W, FAA4/ YMR246W, TAT2/ YOL020W, DIP5/ YPL265W*
15711	organic anion transport	15 out of 140 genes, 10.7%	100 out of 4737 background genes, 2.1%	8.37E-05	0.00	0.00	*CTP1/ YBR291C, VBA2/ YBR293W, AGP1/ YCL025C, YCF1/ YDR135C, GNP1/ YDR508C, HNM1/ YGL077C, MPC3/ YGR243W, MUP3/ YHL036W, FAT3/ YKL187C, GAP1/ YKR039W, YCT1/ YLL055W, FAA4/ YMR246W,*

							TAT2/ YOL020W, ENB1/ YOL158C, DIP5/ YPL265W
6811	ion transport	22 out of 140 genes, 15.7%	215 out of 4737 background genes, 4.5%	0.00011	0.00	0.00	*CTP1/ YBR291C, VBA2/ YBR293W, SUL1/ YBR294W, PHO89/ YBR296C, AGP1/ YCL025C, YCF1/ YDR135C, GNP1/ YDR508C, HNM1/ YGL077C, MEP1/ YGR121C, MPC3/ YGR243W, DUR3/ YHL016C, MUP3/ YHL036W, QDR2/ YIL121W, FAT3/ YKL187C, GAP1/ YKR039W, YCT1/ YLL055W, ATR1/ YML116W, FET3/ YMR058W, FAA4/ YMR246W, TAT2/ YOL020W, ENB1/ YOL158C, DIP5/ YPL265W*
43605	cellular amide catabolic process	5 out of 140 genes, 3.6%	9 out of 4737 background genes, 0.2%	0.00126	0.00	0.00	*DUR3/ YHL016C, DAL1/ YIR027C, DAL2/ YIR029W, DAL7/ YIR031C, DAL3/ YIR032C*
71705	nitrogen compound transport	18 out of 140 genes, 12.9%	192 out of 4737 background genes, 4.1%	0.00564	0.00	0.02	*FUR4/ YBR021W, VBA2/ YBR293W, AGP1/ YCL025C, YCF1/ YDR135C, GNP1/ YDR508C, FCY2/ YER056C, HNM1/ YGL077C, YGL114W, UTP22/ YGR090W, MEP1/ YGR121C, DUR3/ YHL016C, MUP3/ YHL036W, GAP1/ YKR039W, PTR2/ YKR093W, YCT1/ YLL055W, MMP1/ YLL061W, TAT2/ YOL020W, DIP5/ YPL265W*
6865	amino acid transport	8 out of 140 genes, 5.7%	39 out of 4737 background genes, 0.8%	0.00708	0.00	0.02	*VBA2/ YBR293W, AGP1/ YCL025C, GNP1/ YDR508C, MUP3/ YHL036W, GAP1/ YKR039W, YCT1/ YLL055W, TAT2/ YOL020W, DIP5/ YPL265W*

Gene Ontology Enrichment, down-regulated genes

**Gene Ontology ID**	**Gene Ontology term**	**Cluster frequency**	**Background frequency**	**p-value**	**FDR**	**Expected False Positives**	**Genes annotated to the term**
42723	thiamine-containing compound metabolic process	4 out of 109 genes, 3.7%	5 out of 4737 background genes, 0.1%	0.00055	0.02	0.02	*THI2/ YBR240C, THI4/ YGR144W, THI20/ YOL055C, THI21/ YPL258C*
42724	thiamine-containing compound biosynthetic	4 out of 109 genes, 3.7%	5 out of 4737 background genes, 0.1%	0.00055	0.01	0.02	*THI2/ YBR240C, THI4/ YGR144W, THI20/ YOL055C, THI21/ YPL258C*

	process						
6772	thiamine metabolic process	4 out of 109 genes, 3.7%	5 out of 4737 background genes, 0.1%	0.00055	0.01	0.02	*THI2/ YBR240C, THI4/ YGR144W, THI20/ YOL055C, THI21/ YPL258C*
9228	thiamine biosynthetic process	4 out of 109 genes, 3.7%	5 out of 4737 background genes, 0.1%	0.00055	0.01	0.02	*THI2/ YBR240C, THI4/ YGR144W, THI20/ YOL055C, THI21/ YPL258C*
