# Supplementary material for: Genetic variation and expression changes associated with molybdate resistance from a glutathione producing wine strain of Saccharomyces cerevisiae
Source: PLoS One. 2017 Jul 6;12(7):e0180814. doi: 10.1371/journal.pone.0180814 (PMC5500363; doi:10.1371/journal.pone.0180814)
Supplement: S5 Table — Comparison between gene expression levels of the UMCC 2581 and UMCC 855 strains. Gene Ontology (GO) function enriched for up-regulated and down-regulated genes in UMCC 2581 are reported. (PDF) [file pone.0180814.s007.pdf]

S5 Table. Gene Ontology - Function.
Comparison between gene expression levels of the UMCC 2581 and UMCC 855 strains. Gene Ontology (GO) function enriched for up-regulated and down-regulated genes in UMCC 2581 are reported.

Function Ontology

Gene Ontology Enrichment, up-regulated genes

Gene Ontology ID	Gene Ontology term	Cluster frequency	Background frequency	p-value	FDR	Expected False Positives	Genes annotated to the term
8509	anion transmembrane transporter activity	16 out of 140 genes, 11.4%	75 out of 4737 background genes, 1.6%	3.68E-08	0.00	0.00	*CTP1/YBR291C, VBA2/YBR293W, SUL1/YBR294W, PHO89/YBR296C, AGP1/YCL025C, YCF1/YDR135C, GNP1/YDR508C, HNM1/YGL077C, MPC3/YGR243W, MUP3/YHL036W, GAP1/YKR039W, YCT1/YLL055W, MMP1/YLL061W, ATR1/YML116W, TAT2/YOL020W, DIP5/YPL265W*
46943	carboxylic acid transmembrane transporter activity	13 out of 140 genes, 9.3%	50 out of 4737 background genes, 1.1%	1.38E-07	0.00	0.00	*CTP1/YBR291C, VBA2/YBR293W, AGP1/YCL025C, YCF1/YDR135C, GNP1/YDR508C, HNM1/YGL077C, MPC3/YGR243W, MUP3/YHL036W, GAP1/YKR039W, YCT1/YLL055W, MMP1/YLL061W, TAT2/YOL020W, DIP5/YPL265W*
5342	organic acid transmembrane transporter activity	13 out of 140 genes, 9.3%	53 out of 4737 background genes, 1.1%	3.05E-07	0.00	0.00	*CTP1/YBR291C, VBA2/YBR293W, AGP1/YCL025C, YCF1/YDR135C, GNP1/YDR508C, HNM1/YGL077C, MPC3/YGR243W, MUP3/YHL036W, GAP1/YKR039W, YCT1/YLL055W, MMP1/YLL061W, TAT2/YOL020W, DIP5/YPL265W*
8514	organic anion transmembrane transporter activity	13 out of 140 genes, 9.3%	60 out of 4737 background genes, 1.3%	1.57E-06	0.00	0.00	*CTP1/YBR291C, VBA2/YBR293W, AGP1/YCL025C, YCF1/YDR135C, GNP1/YDR508C, HNM1/YGL077C, MPC3/YGR243W, MUP3/YHL036W, GAP1/YKR039W, YCT1/YLL055W, MMP1/YLL061W, TAT2/YOL020W, DIP5/YPL265W*

22891	substrate-specific transmembrane transporter activity	27 out of 140 genes, 19.3%	271 out of 4737 background genes, 5.7%	1.72E-06	0.00	0.00	*FUR4/YBR021W, CTP1/YBR291C, VBA2/YBR293W, SUL1/YBR294W, PHO89/YBR296C, AGP1/YCL025C, YCF1/YDR135C, HXT7/YDR342C, GNP1/YDR508C, STL1/YDR536W, FCY2/YER056C, HNM1/YGL077C, MEP1/YGR121C, MPC3/YGR243W, DUR3/YHL016C, MUP3/YHL036W, HXT5/YHR096C, QDR2/YIL121W, GAP1/YKR039W, YCT1/YLL055W, MMP1/YLL061W, ATR1/YML116W, HXT2/YMR011W, FET3/YMR058W, TAT2/YOL020W, ENB1/YOL158C, DIP5/YPL265W*
15075	ion transmembrane transporter activity	23 out of 140 genes, 16.4%	203 out of 4737 background genes, 4.3%	2.13E-06	0.00	0.00	*FUR4/YBR021W, CTP1/YBR291C, VBA2/YBR293W, SUL1/YBR294W, PHO89/YBR296C, AGP1/YCL025C, YCF1/YDR135C, GNP1/YDR508C, STL1/YDR536W, HNM1/YGL077C, MEP1/YGR121C, MPC3/YGR243W, DUR3/YHL016C, MUP3/YHL036W, QDR2/YIL121W, GAP1/YKR039W, YCT1/YLL055W, MMP1/YLL061W, ATR1/YML116W, FET3/YMR058W, TAT2/YOL020W, ENB1/YOL158C, DIP5/YPL265W*
22857	transmembrane transporter activity	28 out of 140 genes, 20.0%	296 out of 4737 background genes, 6.2%	2.81E-06	0.00	0.00	*FUR4/YBR021W, CTP1/YBR291C, VBA2/YBR293W, SUL1/YBR294W, PHO89/YBR296C, AGP1/YCL025C, SNQ2/YDR011W, YCF1/YDR135C, HXT7/YDR342C, GNP1/YDR508C, STL1/YDR536W, FCY2/YER056C, HNM1/YGL077C, MEP1/YGR121C, MPC3/YGR243W, DUR3/YHL016C, MUP3/YHL036W, HXT5/YHR096C, QDR2/YIL121W, GAP1/YKR039W, YCT1/YLL055W, MMP1/YLL061W, ATR1/YML116W, HXT2/YMR011W, FET3/YMR058W, TAT2/YOL020W, ENB1/YOL158C, DIP5/YPL265W*
22892	substrate-specific transporter activity	29 out of 140 genes, 20.7%	316 out of 4737 background genes, 6.7%	2.98E-06	0.00	0.00	*FUR4/YBR021W, CTP1/YBR291C, VBA2/YBR293W, SUL1/YBR294W, PHO89/YBR296C, AGP1/YCL025C, YCF1/YDR135C, HXT7/YDR342C, GNP1/YDR508C, STL1/YDR536W, FCY2/YER056C, HNM1/YGL077C, YGL114W, MEP1/YGR121C, MPC3/YGR243W, DUR3/YHL016C, MUP3/YHL036W, HXT5/YHR096C, QDR2/YIL121W, GAP1/YKR039W, PTR2/YKR093W, YCT1/YLL055W, MMP1/YLL061W, ATR1/YML116W,*

							HXT2/YMR011W, FET3/YMR058W, TAT2/YOL020W, ENB1/YOL158C, DIP5/YPL265W
5215	transporter activity	31 out of 140 genes, 22.1%	370 out of 4737 background genes, 7.8%	7.69E-06	0.00	0.00	*FLR1/YBR008C, FUR4/YBR021W, CTP1/YBR291C, VBA2/YBR293W, SUL1/YBR294W, PHO89/YBR296C, AGP1/YCL025C, SNQ2/YDR011W, YCF1/YDR135C, HXT7/YDR342C, GNP1/YDR508C, STL1/YDR536W, FCY2/YER056C, HNM1/YGL077C, YGL114W, MEP1/YGR121C, MPC3/YGR243W, DUR3/YHL016C, MUP3/YHL036W, HXT5/YHR096C, QDR2/YIL121W, GAP1/YKR039W, PTR2/YKR093W, YCT1/YLL055W, MMP1/YLL061W, ATR1/YML116W, HXT2/YMR011W, FET3/YMR058W, TAT2/YOL020W, ENB1/YOL158C, DIP5/YPL265W*
15171	amino acid transmembrane transporter activity	9 out of 140 genes, 6.4%	32 out of 4737 background genes, 0.7%	2.78E-05	0.00	0.00	*VBA2/YBR293W, AGP1/YCL025C, GNP1/YDR508C, MUP3/YHL036W, GAP1/YKR039W, YCT1/YLL055W, MMP1/YLL061W, TAT2/YOL020W, DIP5/YPL265W*
8324	cation transmembrane transporter activity	15 out of 140 genes, 10.7%	145 out of 4737 background genes, 3.1%	0.00255	0.00	0.00	*FUR4/YBR021W, PHO89/YBR296C, AGP1/YCL025C, GNP1/YDR508C, STL1/YDR536W, HNM1/YGL077C, MEP1/YGR121C, DUR3/YHL016C, MUP3/YHL036W, QDR2/YIL121W, GAP1/YKR039W, YCT1/YLL055W, FET3/YMR058W, TAT2/YOL020W, ENB1/YOL158C*
15179	L-amino acid transmembrane transporter activity	5 out of 140 genes, 3.6%	16 out of 4737 background genes, 0.3%	0.00938	0.00	0.00	*AGP1/YCL025C, GNP1/YDR508C, MUP3/YHL036W, GAP1/YKR039W, TAT2/YOL020W*

Gene Ontology Enrichment, down-regulated genes, No significant term
