# Supplementary material for: Genetic variation and expression changes associated with molybdate resistance from a glutathione producing wine strain of Saccharomyces cerevisiae
Source: PLoS One. 2017 Jul 6;12(7):e0180814. doi: 10.1371/journal.pone.0180814 (PMC5500363; doi:10.1371/journal.pone.0180814)
Supplement: S6 Table — Comparison between gene expression levels of the UMCC 2581 and UMCC 855 strains. Gene Ontology (GO) component enriched for up-regulated and down-regulated genes in UMCC 2581 are reported. (PDF) [file pone.0180814.s008.pdf]

S6 Table. Gene Ontology – Component.

Comparison between gene expression levels of the UMCC 2581 and UMCC 855 strains. Gene Ontology (GO) component enriched for up-regulated and down-regulated genes in UMCC 2581 are reported.

Component Ontology

Gene Ontology Enrichment, up-regulated genes

Gene Ontology ID	Gene Ontology term	Cluster frequency	Background frequency	p-value	FDR	Expected False Positives	Genes annotated to the term
71944	cell periphery	42 out of 140 genes, 30.0%	481 out of 4737 background genes, 10.2%	3.21E-09	0.00	0.00	*FLR1/ YBR008C, FUR4/ YBR021W, YPC1/ YBR183W, SUL1/ YBR294W, PHO89/ YBR296C, AGP1/ YCL025C, HSP30/ YCR021C, KNH1/ YDL049C, FMP45/ YDL222C, SNQ2/ YDR011W, HXT7/ YDR342C, GNP1/ YDR508C, FIT1/ YDR534C, STL1/ YDR536W, UTR2/ YEL040W, FCY2/ YER056C, STE2/ YFL026W, HNM1/ YGL077C, YGL114W, MTL1/ YGR023W, MEP1/ YGR121C, DUR3/ YHL016C, MYO1/ YHR023W, HXT5/ YHR096C, QDR2/ YIL121W, PRY3/ YJL078C, GAP1/ YKR039W, PTR2/ YKR093W, MMP1/ YLL061W, INA1/ YLR413W, PUN1/ YLR414C, ATR1/ YML116W, HXT2/ YMR011W, FET3/ YMR058W, YNL194C, AGA1/ YNR044W, TAT2/ YOL020W, LDS2/ YOL047C, ENB1/ YOL158C, FIT2/ YOR382W, DIP5/ YPL265W, NCE102/ YPR149W*
5886	plasma membrane	33 out of 140 genes, 23.6%	327 out of 4737 background genes, 6.9%	1.70E-08	0.00	0.00	*FLR1/ YBR008C, FUR4/ YBR021W, SUL1/ YBR294W, PHO89/ YBR296C, AGP1/ YCL025C, HSP30/ YCR021C, FMP45/ YDL222C, SNQ2/ YDR011W, HXT7/ YDR342C, GNP1/ YDR508C, STL1/ YDR536W, FCY2/ YER056C, STE2/ YFL026W, HNM1/ YGL077C, YGL114W, MTL1/ YGR023W, MEP1/ YGR121C, DUR3/ YHL016C, HXT5/ YHR096C, QDR2/ YIL121W, GAP1/ YKR039W, PTR2/ YKR093W, MMP1/ YLL061W, INA1/ YLR413W, PUN1/*

							YLR414C, ATR1/ YML116W, HXT2/ YMR011W, FET3/ YMR058W, YNL194C, TAT2/ YOL020W, ENB1/ YOL158C, DIP5/ YPL265W, NCE102/ YPR149W
16021	integral component of membrane	45 out of 140 genes, 32.1%	817 out of 4737 background genes, 17.2%	0.00105	0.00	0.00	*FLR1/ YBR008C, FUR4/ YBR021W, YPC1/ YBR183W, CTP1/ YBR291C, VBA2/ YBR293W, SUL1/ YBR294W, PHO89/ YBR296C, AGP1/ YCL025C, HSP30/ YCR021C, FMP45/ YDL222C, SNQ2/ YDR011W, YCF1/ YDR135C, HXT7/ YDR342C, GNP1/ YDR508C, STL1/ YDR536W, FCY2/ YER056C, STE2/ YFL026W, HNM1/ YGL077C, YGL114W, MTL1/ YGR023W, MEP1/ YGR121C, DUR3/ YHL016C, MUP3/ YHL036W, HXT5/ YHR096C, QDR2/ YIL121W, GUT2/ YIL155C, YJR124C, FAT3/ YKL187C, GAP1/ YKR039W, PTR2/ YKR093W, YCT1/ YLL055W, MMP1/ YLL061W, INA1/ YLR413W, PUN1/ YLR414C, ATR1/ YML116W, HXT2/ YMR011W, FET3/ YMR058W, YNL194C, YOL013W-A, TAT2/ YOL020W, LDS2/ YOL047C, PHM7/ YOL084W, ENB1/ YOL158C, DIP5/ YPL265W, NCE102/ YPR149W*
31224	intrinsic component of membrane	45 out of 140 genes, 32.1%	825 out of 4737 background genes, 17.4%	0.00137	0.00	0.00	*FLR1/ YBR008C, FUR4/ YBR021W, YPC1/ YBR183W, CTP1/ YBR291C, VBA2/ YBR293W, SUL1/ YBR294W, PHO89/ YBR296C, AGP1/ YCL025C, HSP30/ YCR021C, FMP45/ YDL222C, SNQ2/ YDR011W, YCF1/ YDR135C, HXT7/ YDR342C, GNP1/ YDR508C, STL1/ YDR536W, FCY2/ YER056C, STE2/ YFL026W, HNM1/ YGL077C, YGL114W, MTL1/ YGR023W, MEP1/ YGR121C, DUR3/ YHL016C, MUP3/ YHL036W, HXT5/ YHR096C, QDR2/ YIL121W, GUT2/ YIL155C, YJR124C, FAT3/ YKL187C, GAP1/ YKR039W, PTR2/ YKR093W, YCT1/ YLL055W, MMP1/ YLL061W, INA1/ YLR413W, PUN1/ YLR414C, ATR1/ YML116W, HXT2/ YMR011W, FET3/ YMR058W, YNL194C, YOL013W-A, TAT2/ YOL020W, LDS2/ YOL047C, PHM7/ YOL084W, ENB1/ YOL158C, DIP5/ YPL265W, NCE102/ YPR149W*

Gene Ontology Enrichment, down-regulated genes, No significant term
