# Supplementary material for: Genetic variation and expression changes associated with molybdate resistance from a glutathione producing wine strain of Saccharomyces cerevisiae
Source: PLoS One. 2017 Jul 6;12(7):e0180814. doi: 10.1371/journal.pone.0180814 (PMC5500363; doi:10.1371/journal.pone.0180814)
Supplement: S7 Table — Ref. Allele, reference allele as in S288c reference sequence. SNPs or InDels are considered heterozygous with the calculated allele frequency between 0.25–0.75 and coverage greater than 20. Predicted effect describes the variant using HGVS notation (http://www.hgvs.org/mutnomen/). Genes potentially relevant for the resistant phenotype are indicated in bold. (PDF) [file pone.0180814.s009.pdf]

S7 Table. Genome locations and significant genomic variation of genes found in QTL peak in chromosome 12.
Ref. Allele, reference allele as in S288c reference sequence. SNPs or InDels are considered heterozygous with the calculated allele frequency between 0.25-0.75 and coverage greater than 20. Predicted effect describes the variant using HGVS notation (http://www.hgvs.org/mutnomen/). Genes potentially relevant for the resistant phenotype are indicated in bold.

**Chromosome 12, Peak**									
					**Genomic Variation**				
**Systematic gene name**	**Standard gene name**	**End position**	**Start position**	**Gene description**	**Position**	**Ref. Allele**	**UMCC 855 alleles**	**UMCC 2581 alleles**	**Predicted effect**
YLR187W	*SKG3*	524865	527945	Protein of unknown function	526670	C	C/T	T/T	Synonymous, p.Asp602Asp
					526903	C	C/T	C/C	Missense, p.Thr680Ile
**YLR188W**	***MDL1***	528300	530387	Mitochondrial inner membrane half-type ABC transporter	No heterozygous variations detected in the UMCC 855 CDS				
YLR189C	*ATG26*	534393	530797	UDP-glucose:sterol glucosyltransferase	531565	A	A/G	G/G	Synonymous, p.Ser943Ser
					531589	A	A/G	G/G	Synonymous, p.Asn935Asn
					531796	T	T/A	T/T	Synonymous, p.Arg866Arg
					532255	A	A/G	A/A	Synonymous, p.Ile713Ile
					533249	G	G/T	T/T	Missense, p.Ala382Asp
YLR190W	*MMR1*	535214	536689	Phosphorylated protein of the mitochondrial outer membrane	535748	G	G/A	A/A	Missense, p.Glu179Lys
					535848	T	T/C	C/C	Missense, p.Val212Ala

					535968	T	T/C	T/T	Missense, p.Val252Ala
YLR191W	*PEX13*	537272	538432	Peroxisomal importomer complex component	537571	A	A/G	A/A	Missense, p.Ile100Met
					537622	C	C/G	G/G	Missense, p.Asn117Lys
					538121	A	A/G	A/A	Missense, p.Thr284Ala
					538246	A	A/G	A/A	Synonymous, p.Glu325Glu
					538270	G	G/A	G/G	Synonymous, p.Leu333Leu
					538303	G	G/C	G/G	Synonymous, p.Gly344Gly
YLR192C	*HCR1*	539589	538792	eIF3j component of translation initiation factor 3 (eIF3)	538843	G	G/A	G/G	Synonymous, p.Phe249Phe
					539575	G	G/C	C/C	Missense, p.Asp5Glu
YLR193C	*UPS1*	540536	540009	Phosphatidic acid transfer protein	No heterozygous variations detected in the UMCC 855 CDS				
YLR194C		541573	540809	Structural constituent of the cell wall	540935	A	A/G	A/A	Synonymous, p.Thr213Thr
YLR195C	*NMT1*	543304	541937	N-myristoyl transferase	542794	A	A/G	G/G	Synonymous, p.Leu171Leu
YLR196W	*PWP1*	543968	545698	Protein with WD-40 repeats involved in rRNA processing	544057	T	T/C	C/C	Synonymous, p.Ile30Ile
					544246	G	G/A	A/A	Synonymous, p.Glu93Glu
					544316	A	A/G	G/G	Missense, p.Lys117Glu
					544705	T	T/C	C/C	Synonymous, p.Cys246Cys
					544924	T	T/C	C/C	Synonymous, p.Gly319Gly

					544933	C	C/T	T/T	Synonymous, p.Ala322Ala
					544939	C	C/T	T/T	Synonymous, p.Ser324Ser
					545023	T	T/C	C/C	Synonymous, p.Tyr352Tyr
					545092	C	C/T	T/T	Synonymous, p.Ala375Ala
					545104	G	G/A	A/A	Synonymous, p.Glu379Glu
					545130	G	G/A	A/A	Missense, p.Ser388Asn
					545221	G	G/A	A/A	Synonymous, p.Leu418Leu
					545338	T	T/A	A/A	Missense, p.Asp457Glu
					545416	T	T/C	C/C	Synonymous, p.Ala483Ala
					545525	C	C/T	T/T	Synonymous, p.Leu520Leu
YLR197W	*NOP56*	546097	547611	Essential evolutionarily-conserved nucleolar protein	546249	A	A/C	C/C	Synonymous, p.Val51Val
					546382	A	A/G	G/G	Missense, p.Ile96Val
					546454	T	T/C	C/C	Missense, p.Ser120Pro
					547532	A	A/G	G/G	Missense, p.Glu479Gly
YLR198C		547645	547286	Dubious open reading frame	547532	A	A/G	G/G	Synonymous, p.Phe38Phe
YLR199C	*PBA1*	548769	547853	Protein involved in 20S proteasome assembly	No heterozygous variations detected in the UMCC 855 CDS				
YLR200W	*YKE2*	549012	549356	Subunit of the heterohexameric Gim/prefoldin protein	No heterozygous variations detected in the UMCC 855 CDS				

				complex					
YLR201C	*COQ9*	550293	549511	Protein required for ubiquinone biosynthesis and respiratory growth	No heterozygous variations detected in the UMCC 855 CDS				
YLR202C		550636	550194	Dubious open reading frame	550400	T	T/C	C/C	Stop retained, p.Ter40Ter
					550474	T	T/G	G/G	Intron variant
YLR203C	*MSS51*	551960	550650	Specific translational activator for the mitochondrial COX1 mRNA	550837	G	G/A	A/A	Missense, p.Thr375Met
					551145	G	G/A	A/A	Synonymous, p.Phe272Phe
YLR204W	*QRI5*	552270	552605	Mitochondrial inner membrane protein	No heterozygous variations detected in the UMCC 855 CDS				
**YLR205C**	***HMX1***	553677	552724	ER localized heme oxygenase	552741	A	A/AAG	A/A	Frameshift, p.Phe313fs
					553009	G	G/C	C/C	Synonymous, p.Ala223Ala
					553350	A	A/G	A/A	Missense, p.Tyr110His
YLR206W	*ENT2*	554578	556419	Epsin-like protein required for endocytosis and actin patch assembly	554867	A	A/AT	A/A	Frameshift, p.Glu97fs
YLR207W	*HRD3*	556788	559289	ER membrane protein that plays a central role in ERAD	558995	T	T/C	T/T	Synonymous, p.Ser736Ser
					559129	G	G/A	A/A	Missense, p.Ser781Asn
					559156	G	G/C	G/G	Missense, p.Arg790Pro
YLR208W	*SEC13*	559551	560444	Structural component of 3 complexes	559835	T	T/C	T/T	Synonymous, p.His95His
					560024	C	C/T	C/C	Synonymous, p.Ile158Ile
YLR209C	*PNP1*	561732	560797	Purine nucleoside phosphorylase	No heterozygous variations detected in the UMCC 855 CDS				

YLR210W	*CLB4*	562008	563390	B-type cyclin involved in cell cycle progression	562794	C	C/T	C/C	Synonymous, p.Leu263Leu
YLR211C	*ATG38*	564531	563792	Homodimeric subunit of autophagy-specific PtdIns-3-kinase complex I	No heterozygous variations detected in the UMCC 855 CDS				
YLR212C	TUB4	566281	564860	Gamma-tubulin	565534	A	A/C	C/C	Missense, p.Ser250Ala
					566109	C	C/T	T/T	Missense, p.Ser58Asn
YLR213C	CRR1	567922	566654	Putative glycoside hydrolase of the spore wall envelope	566828	A	A/G	G/G	Synonymous, p.Cys365Cys
					567173	T	T/C	T/T	Synonymous, p.Val250Val
					567254	C	C/T	T/T	Synonymous, p.Leu223Leu
					567751	C	C/T	C/C	Missense, p.Gly58Ser
**YLR214W**	**FRE1**	568567	570627	Ferric reductase and cupric reductase	568580	G	G/A	G/G	Missense, p.Arg5His
					569980	C	C/T	T/T	Missense, p.Arg472Cys
					570032	G	G/A	A/A	Missense, p.Gly489Asp
					570311	T	T/A	A/A	Stop gained, p.Leu582*
YLR215C	CDC123	571858	570776	Protein involved in nutritional control of the cell cycle	570923	T	T/C	T/T	Synonymous, p.Ser312Ser
					571772	T	T/C	C/C	Synonymous, p.Ser29Ser
