# Supplementary material for: Genetic variation and expression changes associated with molybdate resistance from a glutathione producing wine strain of Saccharomyces cerevisiae
Source: PLoS One. 2017 Jul 6;12(7):e0180814. doi: 10.1371/journal.pone.0180814 (PMC5500363; doi:10.1371/journal.pone.0180814)
Supplement: S8 Table — Ref. Allele, reference allele as in S288c reference sequence. SNPs or InDels are considered heterozygous with the calculated allele frequency between 0.25–0.75 and coverage greater than 20. Predicted effect describes the variant using HGVS notation (http://www.hgvs.org/mutnomen/). Genes potentially relevant for the resistant phenotype are indicated in bold. (PDF) [file pone.0180814.s010.pdf]

S8 Table. Genome locations and significant genomic variation of genes found in QTL peak in chromosome 4.
Ref. Allele, reference allele as in S288c reference sequence. SNPs or InDels are considered heterozygous with the calculated allele frequency between 0.25-0.75 and coverage greater than 20. Predicted effect describes the variant using HGVS notation (http://www.hgvs.org/mutnomen/). Genes potentially relevant for the resistant phenotype are indicated in bold.

**Chromosome 4, Peak**									
					**Genomic Variation**				
**Systematic gene name**	**Standard gene name**	**End position**	**Start position**	**Gene description**	**Position**	**Ref. Allele**	**UMCC 855 alleles**	**UMCC 2581 alleles**	**Predicted effect**
YDL018C	*ERP3*	423510	422833	Protein with similarity to Emp24p and Erv25p	423385	T	T/G	G/G	Missense, p.Glu42Asp
YDL017W	*CDC7*	424209	425732	DDK (Dbf4-dependent kinase) catalytic subunit	424226	G	G/A	A/A	Synonymous, p.Lys6Lys
YDL016C		425872	425570	Dubious open reading frame	No heterozygous variations detected in the UMCC 855 CDS				
YDL015C	*TSC13*	426934	426002	Enoyl reductase	426649	C	C/T	T/T	Missense, p.Val96Ile
YDL014W	*NOP1*	427364	428347	Histone glutamine methyltransferase, modifies H2A at Q105 in nucleolus	No heterozygous variations detected in the UMCC 855 CDS				
YDL013W	*SLX5*	429067	430926	Subunit of the Slx5-Slx8 SUMO-targeted ubiquitin ligase (STUbL) complex	No heterozygous variations detected in the UMCC 855 CDS				
YDL012C		431517	431108	Tail-anchored plasma membrane protein with a conserved CYSTM module	431267	T	T/C	T/T	Synonymous, p.Gln55Gln
**YDL010W**	***GRX6***	432330	433025	Cis-golgi localized monothiol glutaredoxin, binds Fe-S cluster	432606	T	T/A	A/A	Missense, p.Ser93Thr

YDL011C		432631	432308	Dubious open reading frame	432606	T	T/A	A/A	Missense, p.Asp9Val
YDL009C		433248	432925	Protein of unknown function	433099	G	G/A	G/G	Synonymous, p.His50His
YDL008W	*APC11*	433497	433994	Catalytic core subunit, Anaphase-Promoting Complex/Cyclosome (APC/C)	433554	A	A/G	A/A	Missense, p.Ser20Gly
YDL007C-A		436824	436567	Putative protein of unknown function	No heterozygous variations detected in the UMCC 855 CDS				
YDL007W	*RPT2*	438047	439360	ATPase of the 19S regulatory particle of the 26S proteasome	No heterozygous variations detected in the UMCC 855 CDS				
YDL006W	*PTC1*	439909	440754	Type 2C protein phosphatase (PP2C)	440001	T	T/C	C/C	Synonymous, p.Phe31Phe
**YDL005C**	***MED2***	442309	441014	Subunit of the RNA polymerase II mediator complex	441267	C	C/ CTGTTGTTGT	CTGTTGTTGT/ CTGTTGTTGT	Inframe insertion, p.Asn345_Asn347dup
					441646	G	G/A	A/A	Missense, p.Pro222Ser
					441647	T	T/C	C/C	Synonymous, p.Gln221Gln
					441788	G	G/T	T/T	Synonymous, p.Ala174Ala
YDL004W	*ATP16*	443029	443511	Delta subunit of the central stalk of mitochondrial F1F0 ATP synthase	No heterozygous variations detected in the UMCC 855 CDS				
YDL003W	*MCD1*	444683	446383	Essential alpha-kleisin subunit of the cohesin complex	445472	G	G/A	A/A	Missense, p.Val264Ile
					445653	A	A/G	A/A	Missense, p.Lys324Arg
					446126	T	T/C	T/T	Missense, p.Ser482Pro

					446188	A	A/G	A/A	Synonymous, p.Val502Val
					446328	G	G/A	G/G	Missense, p.Gly549Glu
YDL002C	*NHP10*	447578	446967	Non-essential INO80 chromatin remodeling complex subunit	No heterozygous variations detected in the UMCC 855 CDS				
YDL001W	*RMD1*	447984	449276	Cytoplasmic protein required for sporulation	448045	C	C/G	G/G	Missense, p.Thr21Arg
					448177	C	C/T	T/T	Missense, p.Ser65Leu
					448388	T	T/C	T/T	Synonymous, p.Thr135Thr
					448634	G	G/C	G/G	Synonymous, p.Gly217Gly
					449024	A	A/G	A/A	Synonymous, p.Pro347Pro
					449208	C	C/T	T/T	Synonymous, p.Leu409Leu
					449237	G	G/A	A/A	Synonymous, p.Val418Val
**YDR001C**	***NTH1***	452475	450220	Neutral trehalase, degrades trehalose	No heterozygous variations detected in the UMCC 855 CDS				
YDR002W	*YRB1*	453045	453650	Ran GTPase binding protein	453381	T	T/G	G/G	Missense, p.Phe113Val
YDR003W	*RCR2*	454122	454754	Vacuolar protein	454751	G	G/A	A/A	Synonymous, p.Lys210Lys
YDR003W-A		454782	454904	Putative protein of unknown function	454817	T	T/C	T/T	Synonymous, p.Ala12Ala
YDR004W	*RAD57*	455201	456583	Protein that stimulates strand exchange	455335	C	C/T	T/T	Synonymous, p.Val45Val
					456230	A	A/C	C/C	Missense, p.Asn344His
					456343	T	T/C	C/C	Synonymous, p.Phe381Phe

					456352	T	T/C	T/T	Synonymous, p.Arg384Arg
YDR005C	*MAF1*	458103	456836	Highly conserved negative regulator of RNA polymerase III	456852	T	T/C	T/T	Missense, p.Glu391Gly
					457671	C	C/T	C/C	Missense, p.Ser118Asn
					458036	G	G/A	G/G	Intron variant
YDR006C	*SOK1*	461247	458542	Protein of unknown function	459909	C	C/T	C/C	Missense, p.Val447Ile
					460050	G	G/A	G/G	Synonymous, p.Leu400Leu
YDR007W	*TRP1*	461842	462516	Phosphoribosylanthranilate isomerase	No heterozygous variations detected in the UMCC 855 CDS				
YDR008C		462602	462252	Dubious open reading frame	No heterozygous variations detected in the UMCC 855 CDS				
YDR009W	*GAL3*	463434	464996	Transcriptional regulator	464012	C	C/T	C/C	Synonymous, p.Ile193Ile
					464025	G	G/A	A/A	Missense, p.Glu198Lys
					464985	T	T/C	T/T	Missense, p.Tyr518His
YDR010C		465383	465051	Dubious open reading frame	465106	G	G/T	G/G	Stop gained, p.Ser93*
					465360	C	C/T	C/C	Stop gained, p.Trp8*
					465382	A	A/G	A/A	Start lost, p.Met1?
YDR011W	*SNQ2*	465919	470424	Plasma membrane ATP-binding cassette (ABC) transporter	467525	C	C/G	G/G	Missense, p.Thr536Ser
					467561	C	C/T	T/T	Missense, p.Ser548Phe
					467803	T	T/A	T/T	Missense, p.Ser629Thr
					468195	A	A/G	G/G	Synonymous, p.Gln759Gln
					469937	G	G/C	C/C	Missense,

									p.Cys1340Ser
					470229	A	A/T	T/T	Synonymous, p.Gly1437Gly
					470286	A	A/G	G/G	Synonymous, p.Gly1456Gly
YDR012W	*RPL4B*	471853	472941	Ribosomal 60S subunit protein L4B	No heterozygous variations detected in the UMCC 855 CDS				
YDR013W	*PSF1*	473157	473783	Subunit of the GINS complex (Sld5p, Psf1p, Psf2p, Psf3p)	No heterozygous variations detected in the UMCC 855 CDS				
YDR014W	*RAD61*	474046	475989	Subunit of a complex that inhibits sister chromatid cohesion	474265	G	G/A	A/A	Missense, p.Ala74Thr
					474320	A	A/G	G/G	Missense, p.Lys92Arg
					474321	A	A/G	G/G	Synonymous, p.Lys92Lys
					474625	T	T/G	G/G	Missense, p.Ser194Ala
					474628	A	A/G	G/G	Missense, p.Ile195Val
					474710	A	A/C	C/C	Missense, p.Asn222Thr
					475248	A	A/G	G/G	Synonymous, p.Gly401Gly
YDR014W-A	*HED1*	477797	478285	Meiosis-specific protein	No heterozygous variations detected in the UMCC 855 CDS				
YDR015C		478199	477960	Dubious open reading frame	No heterozygous variations detected in the UMCC 855 CDS				
YDR016C	*DAD1*	478758	478474	Essential subunit of the Dam1 complex (aka DASH complex)	No heterozygous variations detected in the UMCC 855 CDS				
**YDR017C**	***KCS1***	482267	479115	Inositol hexakisphosphate and inositol heptakisphosphate kinase	480096	G	G/A	A/A	Synonymous, p.Asn724Asn
					480590	A	A/G	G/G	Missense, p.Phe560Leu
					480624	G	G/A	A/A	Synonymous, p.Asn548Asn
					480881	G	G/A	G/G	Missense,

									p.His463Tyr
					481308	C	C/G	G/G	Missense, p.Arg320Ser
YDR018C		483860	482670	Probable membrane protein with three predicted transmembrane domains	No heterozygous variations detected in the UMCC 855 CDS				
**YDR019C**	***GCV1***	485365	484163	T subunit of the mitochondrial glycine decarboxylase complex	484757	G	G/A	G/G	Synonymous, p.Asp203Asp
					485129	A	A/G	G/G	Synonymous, p.Val79Val
YDR020C	*DAS2*	486444	485746	Putative protein of unknown function	No heterozygous variations detected in the UMCC 855 CDS				
YDR021W	*FAL1*	486804	488003	Nucleolar protein required for maturation of 18S rRNA	486871	C	C/T	T/T	Missense, p.Ser23Phe
YDR022C	*ATG31*	488662	488072	Autophagy-specific protein required for autophagosome formation	488205	G	G/A	A/A	Missense, p.Ser153Leu
YDR023W	*SES1*	489508	490896	Cytosolic seryl-tRNA synthetase	490197	G	G/T	T/T	Missense, p.Glu230Asp
YDR024W	*FYV1*	491017	491502	Dubious open reading frame	491144	C	C/T	T/T	Missense, p.Ser43Leu
					491145	A	A/T	T/T	Synonymous, p.Ser43Ser
					491146	G	G/A	A/A	Missense, p.Gly44Arg
YDR025W	*RPS11A*	491515	492324	Protein component of the small (40S) ribosomal subunit	491790	G	G/C	G/G	Intron variant
					491803	C	C/T	T/T	Intron variant
YDR026C	*NSI1*	494268	492556	RNA polymerase I termination factor	492686	T	T/G	G/G	Missense, p.Tyr528Ser
					492752	A	A/T	T/T	Missense, p.Leu506Gln
					492782	C	C/T	C/C	Missense, p.Ser496Asn
					494185	C	C/T	T/T	Synonymous,

									p.Arg28Arg
YDR027C	*VPS54*	497318	494649	Component of the GARP (Golgi-associated retrograde protein) complex	495107	C	C/T	T/T	Missense, p.Asp738Asn
					495483	T	T/C	C/C	Synonymous, p.Leu612Leu
					495642	G	G/T	T/T	Synonymous, p.Leu559Leu
					495757	T	T/C	C/C	Missense, p.Asp521Gly
					495808	A	A/G	G/G	Missense, p.Ile504Thr
					496104	A	A/T	T/T	Synonymous, p.Pro405Pro
					496558	G	G/A	A/A	Missense, p.Ser254Phe
YDR028C	*REG1*	500879	497835	Regulatory subunit of type 1 protein phosphatase Glc7p	499451	C	C/T	T/T	Missense, p.Ala477Thr
					499794	G	G/A	A/A	Synonymous, p.Thr362Thr
YDR029W		501100	501414	Dubious open reading frame	No heterozygous variations detected in the UMCC 855 CDS				
YDR030C	*RAD28*	503273	501753	Protein involved in DNA repair	501804	T	T/C	C/C	Synonymous, p.Gly490Gly
YDR031W	*MIX14*	503498	503863	Mitochondrial intermembrane space protein of unknown function	No heterozygous variations detected in the UMCC 855 CDS				
**YDR032C**	***PST2***	504695	504099	Protein with similarity to a family of flavodoxin-like proteins	504139	A	A/C	C/C	Missense, p.Ile186Ser
					504143	C	C/T	T/T	Missense, p.Glu185Lys
YDR033W	*MRH1*	508147	509109	Protein that localizes primarily to the plasma membrane	No heterozygous variations detected in the UMCC 855 CDS				
YDR034C	*LYS14*	512109	509737	Transcriptional activator involved in regulating lysine biosynthesis	510274	G	G/A	A/A	Synonymous, p.Phe612Phe
					511129	C	C/T	T/T	Synonymous,

								p.Val327Val
				511656	G	G/T	T/T	Synonymous, p.Arg152Arg
YDR034C-A	520692	520516	Putative protein of unknown function	520665	T	T/C	T/T	Missense, p.Ile10Val
